# Supplementary material for: Effectiveness of Interventions and Behaviour Change Techniques for Improving Dietary Intake in Young Adults: A Systematic Review and Meta-Analysis of RCTs
Source: Nutrients. 2019 Apr 11;11(4):825. doi: 10.3390/nu11040825 (PMC6520715; doi:10.3390/nu11040825)
Supplement: Supplementary file 1 [file nutrients-11-00825-s001.zip › Table S1 Final search by database.docx]

Table S1: Final search by database

Medline 1946-present. 26/09/2017

| **#** | **Searches** | **Results** |
| --- | --- | --- |
| 1 | randomized controlled trial/ | 482354 |
| 2 | controlled Clinical Trial/ | 96891 |
| 3 | random*.tw. | 990944 |
| 4 | trial.tw. | 521158 |
| 5 | (control adj group*).tw. | 395724 |
| 6 | 1 or 2 or 3 or 4 or 5 | 1684736 |
| 7 | (young* adj (adult* or men or male* or man or women or female* or woman)).tw. | 157024 |
| 8 | Young Adult/ | 646906 |
| 9 | college aged.tw. | 1091 |
| 10 | ((university or college*) adj student*).tw. | 27903 |
| 11 | 7 or 8 or 9 or 10 | 790386 |
| 12 | Diet/ | 144526 |
| 13 | Healthy Diet/ | 734 |
| 14 | eating/ | 49703 |
| 15 | eating behavio?r.tw. | 5309 |
| 16 | physical activity/ | 90749 |
| 17 | Exercise/ | 90749 |
| 18 | Weight Loss/ | 31993 |
| 19 | obesity/ or obesity, abdominal/ or obesity, metabolically benign/ or obesity, morbid/ | 178652 |
| 20 | Overweight/ | 20346 |
| 21 | 12 or 13 or 14 or 15 or 16 or 17 or 18 or 19 or 20 | 451280 |
| 22 | 6 and 11 and 21 | 8802 |
| 23 | animals/ not (humans/ and animals/) | 4554065 |
| 24 | 22 not 23 | 8725 |
| 25 | addresses/ or lectures/ or anecdotes/ or biography/ or interview/ or comment/ or directory/ or editorial/ or legal cases/ or case reports/ or legislation/ or letter/ or news/ or newspaper article/ or patient education handout/ | 3773732 |
| 26 | 24 not 25 | 8700 |
| 27 | limit 26 to english language | 8463 |

Database(s): **Embase**1947 to present 
Search Strategy: 26/09/2017

| **#** | **Searches** | **Results** |
| --- | --- | --- |
| 1 | randomized controlled trial/ | 473542 |
| 2 | controlled Clinical Trial/ | 449441 |
| 3 | random*.tw. | 1252759 |
| 4 | trial.tw. | 686624 |
| 5 | (control adj group*).tw. | 547266 |
| 6 | 1 or 2 or 3 or 4 or 5 | 2191004 |
| 7 | (young* adj (adult* or men or male* or man or women or female* or woman)).tw. | 202061 |
| 8 | Young Adult/ | 201490 |
| 9 | college aged.tw. | 1206 |
| 10 | ((university or college*) adj student*).tw. | 33041 |
| 11 | 7 or 8 or 9 or 10 | 405389 |
| 12 | diet/ | 228032 |
| 13 | healthy diet/ | 618 |
| 14 | dietary intake/ | 68834 |
| 15 | eating/ | 31502 |
| 16 | eating behavio?r.tw. | 7003 |
| 17 | physical activity/ | 122569 |
| 18 | exercise/ | 255000 |
| 19 | obesity/ or abdominal obesity/ or metabolically benign obesity/ or morbid obesity/ | 377807 |
| 20 | weight reduction/ | 149675 |
| 21 | 12 or 13 or 14 or 15 or 16 or 17 or 18 or 19 or 20 | 1040040 |
| 22 | 6 and 11 and 21 | 5933 |
| 23 | animals/ not (humans/ and animals/) | 1306413 |
| 24 | 22 not 23 | 5913 |
| 25 | addresses/ or lectures/ or anecdotes/ or biography/ or interview/ or comment/ or directory/ or editorial/ or legal cases/ or case reports/ or legislation/ or letter/ or news/ or newspaper article/ or patient education handout/ | 1797928 |
| 26 | 24 not 25 | 5823 |
| 27 | limit 26 to english language | 5636 |

Database(s): **PsycINFO**1806 to September Week 3 2017 
Search Strategy: 26/09/2017

| **#** | **Searches** | **Results** |
| --- | --- | --- |
| 1 | clinical trials/ | 10582 |
| 2 | random*.tw. | 171138 |
| 3 | trial.tw. | 91240 |
| 4 | (control adj group*).tw. | 72012 |
| 5 | 1 or 2 or 3 or 4 | 277406 |
| 6 | (young* adj (adult* or men or male* or man or women or female* or woman)).tw. | 64609 |
| 7 | college aged.tw. | 1158 |
| 8 | ((university or college*) adj student*).tw. | 168498 |
| 9 | 6 or 7 or 8 | 229577 |
| 10 | diets/ | 11370 |
| 11 | eating behavior/ or food intake/ | 22757 |
| 12 | physical activity/ or active living/ or activity level/ | 22959 |
| 13 | EXERCISE/ | 19932 |
| 14 | weight loss/ or weight control/ | 6575 |
| 15 | obesity/ or overweight/ | 22375 |
| 16 | 10 or 11 or 12 or 13 or 14 or 15 | 85775 |
| 17 | 5 and 9 and 16 | 653 |
| 18 | animals/ not (humans/ and animals/) | 6932 |
| 19 | 17 not 18 | 652 |
| 20 | addresses/ or lectures/ or anecdotes/ or biography/ or interview/ or comment/ or directory/ or editorial/ or legal cases/ or case reports/ or legislation/ or letter/ or news/ or newspaper article/ or patient education handout/ | 6018 |
| 21 | 19 not 20 | 652 |
| 22 | limit 21 to english language | 629 |

**Web of Science 26/09/2017**

|  |  |  |
| --- | --- | --- |
| # 15 | [**8,389**](http://apps.webofknowledge.com.ezproxy.newcastle.edu.au/summary.do?product=WOS&doc=1&qid=48&SID=P1lDGAH4ECtbuQkxBVT&search_mode=AdvancedSearch&update_back2search_link_param=yes) | (#14 AND #10 AND #5) *AND***LANGUAGE:** (English) *AND* **DOCUMENT TYPES:** (Article OR Review)  *Indexes=SCI-EXPANDED, SSCI, A&HCI, ESCI Timespan=All years* |
| # 14 | [**1,097,167**](http://apps.webofknowledge.com.ezproxy.newcastle.edu.au/summary.do?product=WOS&doc=1&qid=45&SID=P1lDGAH4ECtbuQkxBVT&search_mode=CombineSearches&update_back2search_link_param=yes) | #13 OR #12 OR #11  *Indexes=SCI-EXPANDED, SSCI, A&HCI, ESCI Timespan=All years* |
| # 13 | [**390,714**](http://apps.webofknowledge.com.ezproxy.newcastle.edu.au/summary.do?product=WOS&doc=1&qid=44&SID=P1lDGAH4ECtbuQkxBVT&search_mode=AdvancedSearch&update_back2search_link_param=yes) | TS=("Exercise" or "Physical activity")  *Indexes=SCI-EXPANDED, SSCI, A&HCI, ESCI Timespan=All years* |
| # 12 | [**501,515**](http://apps.webofknowledge.com.ezproxy.newcastle.edu.au/summary.do?product=WOS&doc=1&qid=42&SID=P1lDGAH4ECtbuQkxBVT&search_mode=AdvancedSearch&update_back2search_link_param=yes) | TS=("Diet" or "nutrition" or "healthy diet" or "eating habits" "eating behavi?r")  *Indexes=SCI-EXPANDED, SSCI, A&HCI, ESCI Timespan=All years* |
| # 11 | [**344,173**](http://apps.webofknowledge.com.ezproxy.newcastle.edu.au/summary.do?product=WOS&doc=1&qid=40&SID=P1lDGAH4ECtbuQkxBVT&search_mode=AdvancedSearch&update_back2search_link_param=yes) | TS= ("Obesity" or "overweight" or "obesity, abdominal", or "weight loss" or "obesity, morbid")  *Indexes=SCI-EXPANDED, SSCI, A&HCI, ESCI Timespan=All years* |
| # 10 | [**599,668**](http://apps.webofknowledge.com.ezproxy.newcastle.edu.au/summary.do?product=WOS&doc=1&qid=39&SID=P1lDGAH4ECtbuQkxBVT&search_mode=AdvancedSearch&update_back2search_link_param=yes) | #9 OR #8 OR #7 OR #6  *Indexes=SCI-EXPANDED, SSCI, A&HCI, ESCI Timespan=All years* |
| # 9 | [**101,873**](http://apps.webofknowledge.com.ezproxy.newcastle.edu.au/summary.do?product=WOS&doc=1&qid=28&SID=P1lDGAH4ECtbuQkxBVT&search_mode=AdvancedSearch&update_back2search_link_param=yes) | TS= ("young adult*")  *Indexes=SCI-EXPANDED, SSCI, A&HCI, ESCI Timespan=All years* |
| # 8 | [**63,104**](http://apps.webofknowledge.com.ezproxy.newcastle.edu.au/summary.do?product=WOS&doc=1&qid=38&SID=P1lDGAH4ECtbuQkxBVT&search_mode=AdvancedSearch&update_back2search_link_param=yes) | TS= ("university student*" or "college student*").  *Indexes=SCI-EXPANDED, SSCI, A&HCI, ESCI Timespan=All years* |
| # 7 | [**446,049**](http://apps.webofknowledge.com.ezproxy.newcastle.edu.au/summary.do?product=WOS&doc=1&qid=36&SID=P1lDGAH4ECtbuQkxBVT&search_mode=AdvancedSearch&update_back2search_link_param=yes) | TS= ("college aged" or "student*")  *Indexes=SCI-EXPANDED, SSCI, A&HCI, ESCI Timespan=All years* |
| # 6 | [**66,623**](http://apps.webofknowledge.com.ezproxy.newcastle.edu.au/summary.do?product=WOS&doc=1&qid=11&SID=P1lDGAH4ECtbuQkxBVT&search_mode=AdvancedSearch&update_back2search_link_param=yes) | TS= ("young men" or "young male*" or "young man" or "young women" or "young female*" or "young woman")  *Indexes=SCI-EXPANDED, SSCI, A&HCI, ESCI Timespan=All years* |
| # 5 | [**2,243,811**](http://apps.webofknowledge.com.ezproxy.newcastle.edu.au/summary.do?product=WOS&doc=1&qid=33&SID=P1lDGAH4ECtbuQkxBVT&search_mode=AdvancedSearch&update_back2search_link_param=yes) | #1 or #2 or #3 or #4  *Indexes=SCI-EXPANDED, SSCI, A&HCI, ESCI Timespan=All years* |
| # 4 | [**1,416,588**](http://apps.webofknowledge.com.ezproxy.newcastle.edu.au/summary.do?product=WOS&doc=1&qid=32&SID=P1lDGAH4ECtbuQkxBVT&search_mode=AdvancedSearch&update_back2search_link_param=yes) | TS=("control group*") OR TS=("trial" or "clinical trial" or "clinical trials" or "controlled trial" or "controlled trials")  *Indexes=SCI-EXPANDED, SSCI, A&HCI, ESCI Timespan=All years* |
| # 3 | [**1,423,660**](http://apps.webofknowledge.com.ezproxy.newcastle.edu.au/summary.do?product=WOS&doc=1&qid=27&SID=P1lDGAH4ECtbuQkxBVT&search_mode=AdvancedSearch&update_back2search_link_param=yes) | TS=("random*")  *Indexes=SCI-EXPANDED, SSCI, A&HCI, ESCI Timespan=All years* |
| # 2 | [**15,964**](http://apps.webofknowledge.com.ezproxy.newcastle.edu.au/summary.do?product=WOS&doc=1&qid=24&SID=P1lDGAH4ECtbuQkxBVT&search_mode=AdvancedSearch&update_back2search_link_param=yes) | TS= ("controlled clinical trial")  *Indexes=SCI-EXPANDED, SSCI, A&HCI, ESCI Timespan=All years* |
| # 1 | [**177,801**](http://apps.webofknowledge.com.ezproxy.newcastle.edu.au/summary.do?product=WOS&doc=1&qid=21&SID=P1lDGAH4ECtbuQkxBVT&search_mode=AdvancedSearch&update_back2search_link_param=yes) | TS=("randomized controlled trial")  *Indexes=SCI-EXPANDED, SSCI, A&HCI, ESCI Timespan=All years* |

| ID | Search | Hits |
| --- | --- | --- |
| #1 | MeSH descriptor: [Controlled Clinical Trials as Topic] this term only | 522 |
| #2 | MeSH descriptor: [Randomized Controlled Trials as Topic] this term only | 21340 |
| #3 | MeSH descriptor: [Randomized Controlled Trial] explode all trees | 171 |
| #4 | (random*):ti,ab,kw | 626390 |
| #5 | (trial):ti,ab,kw | 509406 |
| #6 | (control next group*):ti,ab,kw | 99559 |
| #7 | (#1 or #2 or #3 or #4 or #5 or #6) | 744014 |
| #8 | MeSH descriptor: [Young Adult] this term only | 293 |
| #9 | (young* next (adult* or men or male* or man or women or female* or woman)):ti,ab,kw | 63561 |
| #10 | (college aged):ti,ab,kw | 3051 |
| #11 | ((university or college*) next student*):ti,ab,kw | 2671 |
| #12 | #8 or #9 or #10 or #11 | 68013 |
| #13 | MeSH descriptor: [Diet] explode all trees | 15745 |
| #14 | MeSH descriptor: [Healthy Diet] this term only | 64 |
| #15 | (eating behavio?r):ti,ab,kw | 477 |
| #16 | MeSH descriptor: [Exercise] this term only | 12921 |
| #17 | MeSH descriptor: [Weight Loss] this term only | 4643 |
| #18 | MeSH descriptor: [Obesity] 1 tree(s) exploded | 10355 |
| #19 | MeSH descriptor: [Overweight] this term only | 2676 |
| #20 | (#13 or #14 or #15 or #16 or #18 or #19) | 33532 |
| #21 | (#7 and #12 and #20) | 4175 |
| #22 | (addresses):pt or (lectures):pt or (anecdotes):pt or (biography):pt or (interview):pt or (comment):pt or (directory):pt or (editorial):pt or (legal cases):pt or (case reports):pt or (legislation):pt or (letter):pt or (news):pt or (newspaper article):pt or (patient education handout):pt | 10754 |
| #23 | (#21 not #22) | 4168 |

**Cochrane: 26/09/2017**

**Cinahl 26/09/2017**

| **#** | **Query** | **Results** |
| --- | --- | --- |
| S20 | S6 AND S12 AND S19 | 2,581 |
| S19 | S13 OR S14 OR S15 OR S16 OR S17 OR S18 | 204,818 |
| S18 | (MH "Weight Loss") | 16,494 |
| S17 | (MH "Obesity") OR (MH "Obesity, Morbid") | 64,760 |
| S16 | (MH "Exercise") | 36,648 |
| S15 | (MH "Physical Activity") | 27,574 |
| S14 | (MH "Nutrition") | 21,725 |
| S13 | (MH "Diet+") | 86,589 |
| S12 | S7 OR S8 OR S9 OR S10 OR S11 | 220,149 |
| S11 | TI ((university or college*) N1 student*) or AB ((university or college*) N1 student*) | 13,358 |
| S10 | TI "college aged" OR AB "college aged" | 557 |
| S9 | (MH "Students, College") OR (MH "Students, Undergraduate") OR (MH "Students, Graduate") | 23,572 |
| S8 | (MH "Young Adult") | 163,471 |
| S7 | TI ( (young* N1 (adult* or men or male* or man or women or female or woman)) ) OR AB ( (young* N1 (adult* or men or male* or man or women or female or woman)) ) | 44,420 |
| S6 | S1 OR S2 OR S3 OR S4 OR S5 | 438,702 |
| S5 | TI (control N1 group*) OR AB (control N1 group*) | 66,195 |
| S4 | TI (trial) OR AB (trial) | 214,028 |
| S3 | TI (random*) OR AB (random*) | 231,090 |
| S2 | (MH “randomized controlled trials”) | 59,114 |
| S1 | (MH "clinical trials") | 134,571 |

Update

**Medline 20/09/2017 to 11/10/2018**

| **#** | **Searches** | **Results** |
| --- | --- | --- |
| 1 | randomized controlled trial/ | 469470 |
| 2 | controlled Clinical Trial/ | 92688 |
| 3 | random*.tw. | 1003003 |
| 4 | trial.tw. | 517395 |
| 5 | (control adj group*).tw. | 404389 |
| 6 | 1 or 2 or 3 or 4 or 5 | 1694176 |
| 7 | (young* adj (adult* or men or male* or man or women or female* or woman)).tw. | 159621 |
| 8 | Young Adult/ | 691233 |
| 9 | college aged.tw. | 1107 |
| 10 | ((university or college*) adj student*).tw. | 28850 |
| 11 | 7 or 8 or 9 or 10 | 834568 |
| 12 | Diet/ | 145863 |
| 13 | Healthy Diet/ | 1746 |
| 14 | eating/ | 49542 |
| 15 | eating behavio?r.tw. | 5512 |
| 16 | physical activity/ | 94248 |
| 17 | Exercise/ | 94248 |
| 18 | Weight Loss/ | 32459 |
| 19 | obesity/ or obesity, abdominal/ or obesity, metabolically benign/ or obesity, morbid/ | 181656 |
| 20 | Overweight/ | 21077 |
| 21 | 12 or 13 or 14 or 15 or 16 or 17 or 18 or 19 or 20 | 458926 |
| 22 | 6 and 11 and 21 | 9143 |
| 23 | animals/ not (humans/ and animals/) | 4470149 |
| 24 | 22 not 23 | 9065 |
| 25 | addresses/ or lectures/ or anecdotes/ or biography/ or interview/ or comment/ or directory/ or editorial/ or legal cases/ or case reports/ or legislation/ or letter/ or news/ or newspaper article/ or patient education handout/ | 3777651 |
| 26 | 24 not 25 | 9041 |
| 27 | limit 26 to english language | 8796 |
| 28 | limit 27 to ed=20170920-20181011 | 1241 |

**Embase**  **20/09/2017 to 11/10/2018**

| **#** | **Searches** | **Results** |
| --- | --- | --- |
| 1 | randomized controlled trial/ | 518126 |
| 2 | controlled Clinical Trial/ | 458387 |
| 3 | random*.tw. | 1345971 |
| 4 | trial.tw. | 745479 |
| 5 | (control adj group*).tw. | 585833 |
| 6 | 1 or 2 or 3 or 4 or 5 | 2346020 |
| 7 | (young* adj (adult* or men or male* or man or women or female* or woman)).tw. | 215095 |
| 8 | Young Adult/ | 256081 |
| 9 | college aged.tw. | 1293 |
| 10 | ((university or college*) adj student*).tw. | 35588 |
| 11 | 7 or 8 or 9 or 10 | 467913 |
| 12 | diet/ | 240875 |
| 13 | healthy diet/ | 1705 |
| 14 | dietary intake/ | 71657 |
| 15 | eating/ | 33838 |
| 16 | eating behavio?r.tw. | 7567 |
| 17 | physical activity/ | 132073 |
| 18 | exercise/ | 270790 |
| 19 | obesity/ or abdominal obesity/ or metabolically benign obesity/ or morbid obesity/ | 405050 |
| 20 | weight reduction/ | 151766 |
| 21 | 12 or 13 or 14 or 15 or 16 or 17 or 18 or 19 or 20 | 1103733 |
| 22 | 6 and 11 and 21 | 6962 |
| 23 | animals/ not (humans/ and animals/) | 1323228 |
| 24 | 22 not 23 | 6939 |
| 25 | addresses/ or lectures/ or anecdotes/ or biography/ or interview/ or comment/ or directory/ or editorial/ or legal cases/ or case reports/ or legislation/ or letter/ or news/ or newspaper article/ or patient education handout/ | 1875684 |
| 26 | 24 not 25 | 6840 |
| 27 | limit 26 to english language | 6634 |
| 28 | limit 27 to dd=20170920-20181011 | 773 |

Psychinfo **20/09/2017 to 11/10/2018**

Search Strategy:

| **#** | **Searches** | **Results** |
| --- | --- | --- |
| 1 | clinical trials/ | 11088 |
| 2 | random*.tw. | 182427 |
| 3 | trial.tw. | 97075 |
| 4 | (control adj group*).tw. | 76090 |
| 5 | 1 or 2 or 3 or 4 | 293619 |
| 6 | (young* adj (adult* or men or male* or man or women or female* or woman)).tw. | 69211 |
| 7 | college aged.tw. | 1258 |
| 8 | ((university or college*) adj student*).tw. | 172794 |
| 9 | 6 or 7 or 8 | 238182 |
| 10 | diets/ | 11850 |
| 11 | eating behavior/ or food intake/ | 23848 |
| 12 | physical activity/ or active living/ or activity level/ | 24672 |
| 13 | EXERCISE/ | 20895 |
| 14 | weight loss/ or weight control/ | 6906 |
| 15 | obesity/ or overweight/ | 23643 |
| 16 | 10 or 11 or 12 or 13 or 14 or 15 | 90650 |
| 17 | 5 and 9 and 16 | 709 |
| 18 | animals/ not (humans/ and animals/) | 7142 |
| 19 | 17 not 18 | 708 |
| 20 | addresses/ or lectures/ or anecdotes/ or biography/ or interview/ or comment/ or directory/ or editorial/ or legal cases/ or case reports/ or legislation/ or letter/ or news/ or newspaper article/ or patient education handout/ | 6120 |
| 21 | 19 not 20 | 708 |
| 22 | limit 21 to english language | 685 |
| 23 | limit 22 to up=20170920-20181011 | 51 |

Web of science 2017-2018

| #1 | TS=("randomized controlled trial")  Indexes=SCI-EXPANDED, SSCI, A&HCI, ESCI Timespan=2017-2018 | 39,962 |
| --- | --- | --- |
| #2 | TS= ("controlled clinical trial")  Indexes=SCI-EXPANDED, SSCI, A&HCI, ESCI Timespan=2017-2018 | 2,751 |
|  | TS=("random*")  Indexes=SCI-EXPANDED, SSCI, A&HCI, ESCI Timespan=2017-2018 | 212,369 |
|  | TS=("control group*") OR TS=("trial" or "clinical trial" or "clinical trials" or "controlled trial" or "controlled trials")  Indexes=SCI-EXPANDED, SSCI, A&HCI, ESCI Timespan=2017-2018 | 223,992 |
| #5 | #1 or #2 or #3 or #4  Indexes=SCI-EXPANDED, SSCI, A&HCI, ESCI Timespan=2017-2018 | 329,526 |
| #6 | TS= ("young men" or "young male*" or "young man" or "young women" or "young female*" or "young woman")  Indexes=SCI-EXPANDED, SSCI, A&HCI, ESCI Timespan=2017-2018 | 8,484 |
| #7 | TS= ("college aged" or "student*")  Indexes=SCI-EXPANDED, SSCI, A&HCI, ESCI Timespan=2017-2018 | 85,957 |
| #8 | TS= ("university student*" or "college student*").  Indexes=SCI-EXPANDED, SSCI, A&HCI, ESCI Timespan=2017-2018 | 12,773 |
| #9 | TS= ("young adult*")  Indexes=SCI-EXPANDED, SSCI, A&HCI, ESCI Timespan=2017-2018 | 18,017 |
| #10 | #9 OR #8 OR #7 OR #6  Indexes=SCI-EXPANDED, SSCI, A&HCI, ESCI Timespan=2017-2018 | 109,002 |
| #11 | TS= ("Obesity" or "overweight" or "obesity, abdominal", or "weight loss" or "obesity, morbid")  Indexes=SCI-EXPANDED, SSCI, A&HCI, ESCI Timespan=2017-2018 | 57,207 |
| #12 | TS=("Diet" or "nutrition" or "healthy diet" or "eating habits" "eating behavi?r")  Indexes=SCI-EXPANDED, SSCI, A&HCI, ESCI Timespan=2017-2018 | 65,885 |
| #13 | TS=("Exercise" or "Physical activity")  Indexes=SCI-EXPANDED, SSCI, A&HCI, ESCI Timespan=2017-2018 | 57,155 |
| #14 | #13 OR #12 OR #11  Indexes=SCI-EXPANDED, SSCI, A&HCI, ESCI Timespan=2017-2018 | 153,364 |
| #15 | (#14 AND #10 AND #5) AND LANGUAGE: (English) AND DOCUMENT TYPES: (Article OR Review)  Indexes=SCI-EXPANDED, SSCI, A&HCI, ESCI Timespan=2017-2018 | 1887 |

**Cochrane 2017-2018**

| #1 | MeSH descriptor: [Controlled Clinical Trials as Topic] this term only | 151 |
| --- | --- | --- |
| #2 | MeSH descriptor: [Randomized Controlled Trials as Topic] this term only | 11902 |
| #3 | MeSH descriptor: [Randomized Controlled Trial] explode all trees | 137 |
| #4 | random*:ti,ab,kw | 732515 |
| #5 | trial:ti,ab,kw | 569577 |
| #6 | (control next group*):ti,ab,kw | 123859 |
| #7 | (#1 or #2 or #3 or #4 or #5 or #6) | 857435 |
| #8 | MeSH descriptor: [Young Adult] this term only | 512 |
| #9 | (young* next (adult* or men or male* or man or women or female* or woman)):ti,ab,kw | 72641 |
| #10 | (college aged):ti,ab,kw | 3381 |
| #11 | ((university or college*) next student*):ti,ab,kw | 3035 |
| #12 | #8 or #9 or #10 or #11 | 77528 |
| #13 | MeSH descriptor: [Diet] explode all trees | 16460 |
| #14 | MeSH descriptor: [Healthy Diet] this term only | 217 |
| #15 | (eating behavio?r):ti,ab,kw | 616 |
| #16 | MeSH descriptor: [Exercise] this term only | 13608 |
| #17 | MeSH descriptor: [Weight Loss] this term only | 5136 |
| #18 | MeSH descriptor: [Obesity] 1 tree(s) exploded | 11404 |
| #19 | MeSH descriptor: [Overweight] this term only | 3709 |
| #20 | (#13 or #14 or #15 or #16 or #18 or #19) | 36469 |
| #21 | (#7 and #12 and #20) | 4919 |
| #22 | addresses:pt or lectures:pt or anecdotes:pt or biography:pt or interview:pt or comment:pt or directory:pt or editorial:pt or (legal cases):pt or (case reports):pt or legislation:pt or letter:pt or news:pt or (newspaper article):pt or (patient education handout):pt | 11166 |
| #23 | (#21 not #22) with Cochrane Library publication date Between Sep 2017 and Oct 2018 | 611 |

**Cinahl 01/09/2017 to 31/10/2018**

| **#** | **Query** | **Limiters/Expanders** | **Results** |
| --- | --- | --- | --- |
| S20 | S6 AND S12 AND S19 | Limiters - Published Date: 20170901-20181031  Search modes - Boolean/Phrase | 357 |
| S19 | S13 OR S14 OR S15 OR S16 OR S17 OR S18 | Search modes - Boolean/Phrase | 229,022 |
| S18 | (MH "Weight Loss") | Search modes - Boolean/Phrase | 18,170 |
| S17 | (MH "Obesity") OR (MH "Obesity, Morbid") | Search modes - Boolean/Phrase | 71,190 |
| S16 | (MH "Exercise") | Search modes - Boolean/Phrase | 41,991 |
| S15 | (MH "Physical Activity") | Search modes - Boolean/Phrase | 32,021 |
| S14 | (MH "Nutrition") | Search modes - Boolean/Phrase | 23,259 |
| S13 | (MH "Diet+") | Search modes - Boolean/Phrase | 95,902 |
| S12 | S7 OR S8 OR S9 OR S10 OR S11 | Search modes - Boolean/Phrase | 257,441 |
| S11 | TI ((university or college*) N1 student*) or AB ((university or college*) N1 student*) | Search modes - Boolean/Phrase | 15,464 |
| S10 | TI "college aged" OR AB "college aged" | Search modes - Boolean/Phrase | 631 |
| S9 | (MH "Students, College") OR (MH "Students, Undergraduate") OR (MH "Students, Graduate") | Search modes - Boolean/Phrase | 26,497 |
| S8 | (MH "Young Adult") | Search modes - Boolean/Phrase | 194,419 |
| S7 | TI ( (young* N1 (adult* or men or male* or man or women or female or woman)) ) OR AB ( (young* N1 (adult* or men or male* or man or women or female or woman)) ) | Search modes - Boolean/Phrase | 51,760 |
| S6 | S1 OR S2 OR S3 OR S4 OR S5 | Search modes - Boolean/Phrase | 502,480 |
| S5 | TI (control N1 group*) OR AB (control N1 group*) | Search modes - Boolean/Phrase | 80,339 |
| S4 | TI (trial) OR AB (trial) | Search modes - Boolean/Phrase | 247,716 |
| S3 | TI (random*) OR AB (random*) | Search modes - Boolean/Phrase | 268,846 |
| S2 | (MH “randomized controlled trials”) | Search modes - Boolean/Phrase | 77,623 |
| S1 | (MH "clinical trials") | Search modes - Boolean/Phrase | 143,163 |
