# Supplementary material for: Effectiveness of Interventions and Behaviour Change Techniques for Improving Dietary Intake in Young Adults: A Systematic Review and Meta-Analysis of RCTs
Source: Nutrients. 2019 Apr 11;11(4):825. doi: 10.3390/nu11040825 (PMC6520715; doi:10.3390/nu11040825)
Supplement: Supplementary file 1 [file nutrients-11-00825-s001.zip › Table S2 Summary of included studies.docx]

| **First author, year, study name, citation**  Table S2: Summary of included studies | **N of additional linked papers (citations)** | **Country** | **Setting** | **Study participants (age range; % male)** | ***N* at baseline** | **Delivery mode** | **Intervention (I)/comparator (C)** | **Duration** | **Data collection time points** | **Retention** | **Diet Measure** | **Dietary outcomes*^≠^*** | **Effect (between grp differences)*** |
| --- | --- | --- | --- | --- | --- | --- | --- | --- | --- | --- | --- | --- | --- |
| Allman-Farinelli (2016), TXT2BFIT (1) | 6 (2-7) | Australia | Community | 18-35yrs; 39% male | 250 | eHealth (SMS, Email, Mobile app, website) + Print | I: 18-page booklet, sample meal plans, 5 coaching calls, 96 SMS, 12 emails, apps, and downloadable resources from the study website. Behaviours addressed were intake of fruits, vegetables, SSBs, take-out meals, and physical activity. After 12 weeks, the intervention arm received 2 further coaching calls, 6 SMS, and 6 emails with continued access to the study website during 6-month follow-up.  C: Two-page handout summarizing the Australian national dietary and physical activity guidelines,1 phone call to introduce them to study procedures and 4 text messages. | 3-m | 0, 3-m, 9-m | 3-m: 89.6%, 6-m: 80.8% | Short diet Questions | Fruit (serves/day) | 3-m: 1=2  9-m: 1>2 |
|  |  |  |  |  |  |  |  |  |  |  |  | Veg (Serves/day) | 3-m: 1>2  9-m: 1>2 |
|  |  |  |  |  |  |  |  |  |  |  |  | SSB (weekly intake) | 3-m: 1>2  9-m: 1>2 |
|  |  |  |  |  |  |  |  |  |  |  |  | Frequency of Take-out meals (weekly intake) | 3-m: 1>2  9-m: 1>2 |
| Annesi (2015) (8) | 0 | USA | University | 17-29yrs; 44% male | 98 | F2F + Print | I (arm 1): *Coach Approach–supplemented class with mid-size groups* - same as control + Goal setting, self-monitoring, feedback on behaviour, behavioural contract signed and self-regulatory skills in groups of 15-20  I (arm 2): *Coach Approach–supplemented class with small-size groups* – same as arm 1 but in groups of 8-10  C (arm 3): *Usual class processes* 2 x weekly class meetings of 75 minutes each covered components of fitness, stress management, weight management, cardiovascular disease, and nutrition and was supported by a textbook that overviewed the value of healthy behaviours. | 15-wk | 0, 15-wk | 15-wk: NR | Short diet Questions | Fruit & Veg (serves/day)*^≠^* | 15-wk: 1=2=3 |
| Ashton (2017), HEYMAN (9) | 0 | Australia | Community | 18-25yrs, 100% male | 50 | eHealth (website, social media, app, wearable device) + F2F (group & individual) | I: Gender-targeted program with access to website, F2F (group and individual), jawbone wearable device, portion control tool, Gymstick resistance band, personalised food and nutrient report; a private Facebook discussion group. Behaviours addressed were eating habits, physical activity and stress management.  C: Waitlist-control | 3-m | 0, 3-m | 3-m: 94% | FFQ (Australian Eating Survey) | Diet Quality (ARFS score)***^≠^*** | 3-m: 1=2 |
|  |  |  |  |  |  |  |  |  |  |  |  | Fruit (serves/day) | 3-m: 1=2 |
|  |  |  |  |  |  |  |  |  |  |  |  | Veg (serves/day) | 3-m: 1>2 |
|  |  |  |  |  |  |  |  |  |  |  |  | Energy intake (kJ/day) | 3-m: 1=2 |
|  |  |  |  |  |  |  |  |  |  |  |  | % energy from ED-NP | 3-m: 1>2 |
| Brookie (2017) ‘Let them eat fruit’ (10) | 0 | NZ | University | 18-25yrs, 33% male | 174 | SMS | I (arm 1): Challenged to increase FV consumption to 5 servings/day. Received a $10 voucher for a local greengrocer. Received two diet SMS/ day for 13 days.  I (arm 2): Supplied with a package of 13 servings of fruit and 13 servings of vegetables. Asked to consume one additional serving of fruit and one additional serving of vegetables (2 servings total) every day.  C (arm 3): Provided with a 14-piece packet of sugar-free chewing gum, and were asked to consume one piece a day for the duration of the intervention. | 2-wk | 0, 2-wk | 2-wk: 98% | FFQ (based on NZ Adult Nutrition Survey) | Fruit & Veg (serves/day)***^≠^*** | 2-wk: 1>3, 2>3, 1=2 |
|  |  |  |  |  |  |  |  |  |  |  |  | Hot chips serves/week | 2-wk: 1=2=3 |
|  |  |  |  |  |  |  |  |  |  |  |  | Sweets / lollies serves/week | 2-wk: 1=2=3 |
| Buscemi (2011), (11) | 0 | USA | University | 18-35yrs, 14% male | 70 | F2F + Print + Phone call | I: 1x 50–60-min session to encourage students to increase their PA, monitor portion size, increase intake of fruits and vegetables and decrease consumption of fast food and SSB's in order to decrease body mass index; handout with tips to change diet and exercise; booster phone call at 2-wks.  C: Provided with information about the on-campus recreation centre, web sites that might be helpful for those interested in making changes in diet/exercise, self-monitoring sheets for both diet and exercise, contact information for on-campus dietitian & personal trainer, portion control information, nutrition facts for common fast food and sugar-sweetened beverage items. | 3-m | 0, 3-m | 3-m: 86% | FFQ  (SSB on 5-point scale from 1 = never to  5 = 3+/day. Fast food was on 4-point scale from  1 = <once a month to  4 = >once a week.) | Fruit (serves/day) | 3-m: 1=2 |
|  |  |  |  |  |  |  |  |  |  |  |  | Veg (serves/day) | 3-m: 1=2 |
|  |  |  |  |  |  |  |  |  |  |  |  | Frequency of SSB | 3-m: 1=2 |
|  |  |  |  |  |  |  |  |  |  |  |  | Frequency of fast food | 3-m: 1=2 |
| Chang (2010), ‘Mothers in motion’ (12) | 1 (13) | USA | Community | 18-34yrs, 0% male | 129 | F2F + DVD + print + teleconference | I: Participants were asked to view a designated chapter in the DVD every other week (10-15 minutes/chapter) for 10 weeks + 5 (30-min) peer support group teleconferences at alternate weeks +3 quiz questions and set 1 or 2 personal goal(s) for healthful behavioural changes and weekly worksheet to self-monitor progress for 1 week.  C: Nutrition education for approximately 20 minutes every six months during the re-certification appointment for her young child(ren). | 10-wks | 0, 4.5-m, 10.5-m | 4.5-m: 54% 10.5-m: 37% | Short diet questions (NCI fat screener and NCI F&V short assessment) | Fruit and Veg (cups/day) | 4.5-m: 1=2  10.5-m: 1=2 |
|  |  |  |  |  |  |  |  |  |  |  |  | % of total calories from fat | 4.5-m: 1=2  10.5-m: 1=2 |
| Chapman (2009), (14) | 0 | UK | University | 18-25yrs, 26% male | 557 | F2F | I (arm 1): Provided with message: *"We want you to plan to eat 5 portions of fruit and veg per day during the next week, paying particular attention to the situations in which you will implement these plans…"* Then instructed to write plans using an if-then format.  I (arm 2): Only difference between implementation groups was in the instructions given: no format specified for writing plans.  C (arm 3): Usual lifestyle | Brief 1 session | 0, 1-wk | 1-wk: 54% | Single item F&V question | Fruit & Veg (portions/day)***^≠^*** | 1-wk: 1>3, 2>3 |
| Do (2008) (15) | 1 (16) | USA | Community | 18-24yrs, 39% male | 2024 | Print + telephone | I: Received 6 mailings including 2 individualized stage-tailored reports, 4 colourful stage-tailored for FV intake newsletters, and an F&V Connections magazine over a 6-month period and two educational calls in 6 months.  C: Received a mailed 5-A-Day pamphlet | 6-m | 0, 4-m, 12-m | 4-m: 79% 12-m: 62% | FFQ | Fruit & Veg (serves/day)***^≠^*** | 4-m: 1>2  12-m: 1>2 |
|  |  |  |  |  |  |  |  |  |  |  |  | Fruit (serves/day) | 4-m: 1>2  12-m: 1>2 |
|  |  |  |  |  |  |  |  |  |  |  |  | Veg (serves/day) | 4-m: 1=2  12-m: 1=2 |
| Eiben (2006), ‘Health Hunters’ (17) | 0 | Sweden | Community | 18-28yrs, 0% male | 40 | F2F+ email + telephone | I: Received a customized support package, divided into three main theme areas: physical activity, diet and weight control. Within each theme area, informational and self-help materials were prepared relevant to the expressed needs and preferences of individuals. Also included special interest lectures, and ‘booster’ visits with dietitians.  C: Waitlist control | 12-m | 0, 13-months | 1-yr: 75% | FFQ | Energy intake (kcal/day) | 13-m: 1=2 |
|  |  |  |  |  |  |  |  |  |  |  |  | Fat intake (% energy) | 13-m: 1=2 |
|  |  |  |  |  |  |  |  |  |  |  |  | Carbohydrate intake (%energy) | 13-m: 1=2 |
|  |  |  |  |  |  |  |  |  |  |  |  | Protein intake (%energy) | 13-m: 1=2 |
|  |  |  |  |  |  |  |  |  |  |  |  | Fibre intake (g/1000 kcal) | 13-m: 1=2 |
| Franko (2008). ‘My Student body’ (18) | 0 | USA | University | 18-24yrs, 43% male | 606 | Online | I (arm 1): Instructed to use online nutrition program for two web sessions  I (arm 2): Instructed to use online nutrition program for two web sessions and a subsequent booster session  C (arm 3): Instructed to use an interactive anatomy education website for two web sessions. | 2-wks | 0, 2-wk, 3-m, 6-m | 2-wk: 73%  3-m : 69%  6-m: 70% | FFQ | Fruit & Veg (serves/day)***^≠^*** | 2-wk:1=2=3  3-m:1=2=3, 6-m: 1=2=3 |
|  |  |  |  |  |  |  |  |  |  |  |  | % energy from fat | 2-wk:1=2=3  3-m: 1=2=3, 6-m: 1=2=3 |
|  |  |  |  |  |  |  |  |  |  |  |  | Fibre (g/day) | 2-wk:1=2=3  3-m: 1=2=3, 6-m: 1=2=3 |
|  |  |  |  |  |  |  |  |  |  |  | Single item question | Fruit and veg (serves/day) | 2-wk: 1>3, 2>3, 1=2  3-m: 1=2=3, 6-m: 1=2=3 |
| Franko (2012), (19) | 0 | USA | University | 18-24yrs, 0% male | 64 | Online | I: Participants completed two computer-based programs to improve eating behaviours.  C: Used two non-eating-related websites | 2-wks | 0, 2-wk, 3-m | 2-wk: 92%, 3-m: 80% | Single item question | Fruit and veg (serves/day)***^≠^*** | 2-wk: 1>2,  3-m: 1>2 |
| Goodman (2016), (20) | 0 | Canada | Community | 18-25yrs, 42% male | 109 | Video + mobile app | I: Behavioural intervention consisting of an educational video component and use of handheld mobile app to track vitamin D intake.  C: Wait-list control | 3-m | 0, 3-m | 3-m: 83% | FFQ | Vit D intake from food and drink (IU/day)***^≠^*** | 3-m: 1=2 |
| Gow (2010), (21) | 0 | USA | University | 17-22yrs, 26% male | 159 | Online + email | I: (arm 1): Combined group weight and caloric feedback (via email) occurring within an internet intervention addressing healthy eating, increased physical activity, media literacy and positive body image;  I: (arm 2): Weight and caloric feedback alone (via email)  I: (arm 3): The Internet intervention alone  C: (arm 4): No treatment | 6-wk | 0, 6-wk, 3-m (removed from analysis due to high drop-out) | 6-wk: 69% 3-m: 11% | 27-item Block screener | Fruit & Veg (unit not provided) | 6-wk & 3-m: 1=2=3=4 |
|  |  |  |  |  |  |  |  |  |  |  |  | Fibre (unit not provided) | 6-wk & 3-m: 1=2=3=4 |
|  |  |  |  |  |  |  |  |  |  |  |  | Fat (unit not provided) | 6-wk & 3-m: 1=2=3=4 |
| Greene (2012), ‘Project Webhealth’ (22) | 1 (23) | USA | University | 18-24yrs, 38% male | 2343 | Online | I: 10-lesson web-based nutrition and PA intervention with following topics: body size acceptance, hunger and fullness, physical activity, skills for good nutrition, enhancing food variety, eating enjoyment, and maintaining a healthy weight.  C: Usual lifestyle | 10-wks | 0, 3-m, 15-m | 3-m: 57%  15-m: 68% | 2-item NCI screener for F&V | Fruit & Veg (cups/day)***^≠^*** | 3-m: 1>2  15-m: 1>2 |
| Hebden (2014), (24) | 0 | Australia | University | 18-30yrs, 20% male | 51 | SMS + Email + online + print + mobile app | I: Information booklet (as with control) + 12-week mHealth programme which addressed four key lifestyle behaviours: physical activity, sedentary behaviour, intake of fruit and vegetables, energy-dense takeaway meals and SSB. Received two SMS and two e-mails each week, and access to one smartphone application and one Internet forum.  C: Information booklet covering core food groups, meal plan and exercise guidelines. | 12-wks | 0, 13-wks | 13-wk: 90% | Short diet questions | Fruit & Veg (serves/day) | 13-wk: 1=2 |
|  |  |  |  |  |  |  |  |  |  |  |  | Energy dense takeaway meals (≤ 1 time /week) | 13-wk: 1=2 |
|  |  |  |  |  |  |  |  |  |  |  |  | SSB (ml/week) | 13-wk: 1=2 |
| Heeren (2017) ‘Wake-up!’, (25) | 0 | South Africa | University | 18-24yrs, 47% male | 176 | F2F (group) | I: The health-promotion intervention was designed to increase knowledge, attitudes, self-efficacy, and skills to prevent NCDs by increasing physical activities, choosing healthy diets, and limiting alcohol use.  C: Attention matched control focused on HIV risk. | 8-wks | 0, 6-m, 12-m | 6-m: 97%, 12-m: 97% | 7-item FFQ (NCI) | Fruit (serves/day) | 6-m: 1=2,  12-m :1=2 |
|  |  |  |  |  |  |  |  |  |  |  |  | Veg (serves/day) | 6-m: 1=2,  12-m: 1=2 |
|  |  |  |  |  |  |  |  |  |  |  |  | Fried food (serves/day) | 6-m: 1=2,  12-m: 1=2 |
|  |  |  |  |  |  |  |  |  |  |  |  | % 5-A-day | 6-m: 1=2,  12-m: 1=2 |
| Hivert (2007) (26) | 0 | Canada | University | 17-26yrs, 18% male | 115 | F2F (group) | I: Small-group interactive seminars of 45 min every 2 weeks for the first 2 months of the academic calendar and every month thereafter for the remaining 2 years (a total of 23 seminars over 2 years). Seminars were designed to help maintain a healthy lifestyle  C: Usual lifestyle | 24-m | 0, 3-m, 6-m, 12-m, 18-m, 24-m | 24-m: 83% | 3- food diaries | Caloric intake (kcal/day) | All time points: 1=2 |
|  |  |  |  |  |  |  |  |  |  |  |  | % CHO | All time points: 1=2 |
|  |  |  |  |  |  |  |  |  |  |  |  | % Protein | All time points: 1=2 |
|  |  |  |  |  |  |  |  |  |  |  |  | % Lipids | All time points: 1=2 |
| Hutchesson (2018) ‘Be Positive Be Health*e’* (27) | 0 | Australia | Community | 18-35yrs, 0% male | 57 | eHealth (website, social media, smartphone application, email, SMS) | I: Received ‘Be Positive Be Health*e’* (BPBH) ehealth program. BPBH supported participants to modify diet and physical activity behaviours using evidenced-based strategies (e.g., self-monitoring) tailored for young women.  C: Waitlist control | 6-m | 0, 6-m | 6-m: 75% | FFQ (Australian Eating Survey) | Energy intake (kJ/day) | 6-m: 1=2 |
|  |  |  |  |  |  |  |  |  |  |  |  | Fruit (% energy/day) | 6-m: 1=2 |
|  |  |  |  |  |  |  |  |  |  |  |  | Fruit (grams/day) | 6-m: 1=2 |
|  |  |  |  |  |  |  |  |  |  |  |  | Veg (% energy/day) | 6-m: 1>2 |
|  |  |  |  |  |  |  |  |  |  |  |  | Veg (grams/day) | 6-m: 1=2 |
|  |  |  |  |  |  |  |  |  |  |  |  | Takeaway (% energy/day) | 6-m: 1=2 |
|  |  |  |  |  |  |  |  |  |  |  |  | % energy from non-core foods | 6-m: 1>2 |
|  |  |  |  |  |  |  |  |  |  |  |  | % energy from core foods | 6-m: 1>2 |
| Jakicic (2016), IDEA (28) | 1 (29) | USA | Community | 18-35yrs, 29% male | 470 | Website + wearable device + F2F (group) + telephone + SMS | I: Same as control but provided with a wearable device and accompanying web interface to monitor diet and physical  C: Active control: Participants were placed on a low-calorie diet, prescribed increases in physical activity, and had group counselling sessions. At 6 months, the interventions added telephone counseling sessions, text message prompts, and access to study materials on a website. At 6 months, initiated self-monitoring of diet and physical activity using a website. | 24-m | 0, 6-m, 12-m, 18-m, 24-m | 24-m: 75% | Diet History Questionnaire | Energy intake (kcal/day) | All time points: 1=2 |
|  |  |  |  |  |  |  |  |  |  |  |  | % Calories from CHO | All time points: 1=2 |
|  |  |  |  |  |  |  |  |  |  |  |  | % Calories from Protein | All time points: 1=2 |
|  |  |  |  |  |  |  |  |  |  |  |  | % Calories from Fat | All time points: 1=2 |
| Jung (2011), (30) | 0 | Canada | University | 18-19yrs, 0% male | 133 | F2F (group) + print | I: 45 min seminar and received targeted, gain-framed materials relating to nutritional information of calcium-rich foods.  C: Received 45 min seminar, as it was considered standard care | 12-m | 0, 1-wk, 2-m, 6-m, 12-m | 1-wk: 100%  2-m: 74% 6-m: 69%  12-m: 74% | 3-day food log | Energy intake (kcal/day) | 1-wk: 1=2  2-m: 1=2  6-m: 1=2  12-m: 1=2 |
|  |  |  |  |  |  |  |  |  |  |  |  | Calcium intake***^≠^*** | 1-wk: 1=2  2-m: 1=2  6-m: 1>2  12-m: 1>2 |
| Kattelmann (2014), Y.E.A.H (31) | 2 (32, 33) | USA | University | 18-24yrs, 33% male | 1639 | F2F (group) + email | I: 21 mini educational lessons and e-mail messages (called nudges). The lessons addressed eating behaviour, physical activity, stress management, and healthy weight management through  a non-diet approach  C: Usual lifestyle | 10-wks | 0, 10-wks, 15-m | 10-wk: 76%  15-m: 59% | NCI Screener | Fruit & Veg (cups/day) | 10-wk: 1>2  15-m: 1=2 |
|  |  |  |  |  |  |  |  |  |  |  |  | % energy from fat | 10-wk: 1>2  15-m: 1>2 |
|  |  |  |  |  |  |  |  |  |  |  |  | SSB (kcal/day) | 10-wk: 1=2  15-m: 1=2 |
|  |  |  |  |  |  |  |  |  |  |  |  | Wholegrain (serves/day) | 10-wk: 1=2  15-m: 1=2 |
| Kendzierski (2015) (34) | 0 | USA | University | Exp 1: 18-28yrs, 25% male | Exp 1: 173 | Print + F2F | I: Implementation intention condition participants listed what fruits and vegetables they would eat and when and where they would eat them.  C: Asked to try to eat at least 2 servings of fruit per day and at least 3 servings of vegetables per day for the next week but no further instruction. | 1-wk | 0, 1-wk | Exp 1:  1-wk: 82% | 14-item FFQ | Fruit (serves/day)***^≠^*** | Exp 1:  1-wk:1>2 |
|  |  |  |  |  |  |  |  |  |  |  |  | Veg (serves/day)***^≠^*** | Exp 1:  1-wk:1>2 |
| Kendzierski (2015) (34) | 0 | USA | University | Exp 2: 18-22yrs, 32% male | Exp 2: 83 | Print + F2F | I: Implementation intention condition participants listed what fruits and vegetables they would eat and when and where they would eat them.  C: Asked to try to eat at least 2 servings of fruit per day and at least 3 servings of vegetables per day for the next week but no further instruction. | 1-wk | 0, 1-wk | Exp 2:  1-wk: 95% | 14-item FFQ | Fruit (serves/day)***^≠^*** | Exp 2:  1-wk:1=2 |
|  |  |  |  |  |  |  |  |  |  |  |  | Veg (serves/day)***^≠^*** | Exp 2:  1-wk:1>2 |
| Kerr (2016), CHAT (35) | 1 (36) | Australia | Community | 18-30yrs, 34% male | 247 | SMS | I: (arm 1): Dietary feedback and weekly text messages  I: (arm 2): Dietary feedback only  C: Usual lifestyle | 6-m | 0, 6-m | 6-m: 89% | Mobile food record | Fruit (serves/day)***^≠^*** | 6-m: 1=3, 2=3 |
|  |  |  |  |  |  |  |  |  |  |  |  | Veg (serves/day)***^≠^*** | 6-m: 1=3, 2=3 |
|  |  |  |  |  |  |  |  |  |  |  |  | SSB (serves/day) | 6-m: 1=3, 2=3 |
|  |  |  |  |  |  |  |  |  |  |  |  | EDNP (serves) | 6-m: 1=3, 2=3 |
| Knauper (2011) (37) | 0 | USA | University | 17-22yrs, 38% male | 247 | F2F | I (arm 1): Implementation intentions  I (arm 2): Goal intention mental imagery  I: (arm 3): Mental imagery targeted to the implementation intentions.  C (arm 4): Goal intention | 1-wk | 0, 1-wk | 1-wk: 96% | Single question | Fruit (portions/day) | 1-wk: 1>4, 2>4, 3>4, 1=2, 3>1, 3>2 |
| Kothe (2012) ‘Fresh Facts’(38) | 0 | Australia | Community | 18-25yrs, 24% male | 194 | Email | I: (High frequency group) 27 intervention emails each containing one intervention message designed to increase fruit and vegetable consumption and based on TPB  C: (low-frequency group) received 9 longer emails. The email content was matched across the two groups so that all participants received identical intervention content regardless of group. | 1-m | 0, 1-m | 1-m: 86% | Short diet questions | Fruit and Veg (serves/day)***^≠^*** | 1-m: 1=2 |
| Kothe (2014) ‘Fresh Facts’ (39) | 1 (40) | Australia | Community | 18-25yrs, 17% male | 162 | Email | I: Received automated email messages promoting fruit and vegetable consumption every 3 days over the course of the 1 month intervention. Messages targeted attitude, subjective norm, and perceived behavioural control.  C: Usual lifestyle | 1-m | 0, 1-m | 1-m: 82% | Short diet questions | Fruit and Veg (serves/day)***^≠^*** | 1-m: 1=2 |
| Kreausukon (2012) (41) | 0 | Thailand | University | 18-25yrs, NR | 121 | F2F (group) | I: Received a training program including general health and nutrition education plus psychological program focusing on self-efficacy enhancement and planning skills.  C: Active control group received only the general health and nutrition education | 1-wk | 0, 1-wk, 6-wks | 6-wk: 94% | 2-item diet questions | Fruit and Veg (serves/day)***^≠^*** | 1-wk: 1>2  6-wk: 1>2 |
| Kypri (2005) (42) | 0 | New Zealand | University | 17-24yrs, 51% male | 218 | Online | I (arm 1): Computerized assessment, feedback and advice on fruit and vegetable consumption, PA, alcohol, and smoking.  I (arm 2): Computerised assessment only  C (arm 3): Minimal contact at baseline | 6-wk | 0, 6-wk | 6-wk: 83% | 2- questions from National survey | Fruit and Veg (% meeting recommendations)***^≠^*** | 6-wk: 1>3, 1=2, 2=3 |
| LaChausse (2012), My Student Body’ (43) | 0 | USA | University | 18-35yrs, 24% male | 358 | Online + F2F (group) | I (arm 1): Online course - interactive, internet-based program designed to provide nutrition and physical fitness education to college students  I (arm 2): An on-Campus course - met once a week for 2 hours for 12 weeks. The course addressed issues in weight management, including stress, basic nutrition and exercise principles, weight-related diseases, eating behaviours, time management, and overall wellness.  C (arm 3): Usual lifestyle | 12-wks | 0, 14-wks | 14-wk: 87% | FFQ | Fruit (frequency/day) | 14-wks: 1>2, 1>3, 2=3 |
|  |  |  |  |  |  |  |  |  |  |  |  | Veg (frequency/day) | 14-wks: 1>2, 1>3, 2=3 |
| Laska (2016), ‘Choices’ (44) | 4 (45-48) | USA | University | 18-35yrs, 32% male | 441 | F2F (group), Social media, website. | I: CHOICES intervention (Choosing Healthy Options in College Environments and Settings) delivered via an academic course and a social network and support website. The intervention focused on  diet/nutrition, physical activity, screen time, and sleep  C: Received health assessments | 24-m | 0, 4-m, 12-m, 24-m | 4-m: 92%, 12-m: 89%  24-m: 83% | NCI Diet History Questionnaire | Fast food (times/week) | 4-m: 1=2  12-m: 1=2  24-m: 1>2 |
|  |  |  |  |  |  |  |  |  |  |  |  | SSB (times/day) | 4-m: 1=2  12-m: 1=2  24-m: 1=2 |
|  |  |  |  |  |  |  |  |  |  |  |  | Breakfast (% eating 5-7 days/wk) | 4-m: 1=2  12-m: 1=2  24-m: 1=2 |
|  |  |  |  |  |  |  |  |  |  |  |  | Weekly meals prepared at home | 4-m: 1=2  12-m: 1=2  24-m: 1=2 |
| Lhakhang (2014), (49) | 0 | India | University | 18-26yrs, 48% male | 224 | F2F (group) | I: Sequence 1 - received a motivational intervention (outcome expectancies, risk perception, and task self-efficacy) for 2-wk followed by a self-regulatory intervention (planning and dietary self-efficacy)    C: Active control Sequence 2: received a self-regulatory intervention (planning and dietary self-efficacy) for 2-wks followed by a motivational intervention (outcome expectancies, risk perception, and task self-efficacy) | 1-m | 0, 2-wks, 1-m | 2-wk: 92%  1-m: 92% | Short diet questions | Fruit and Veg (portions/day)***^≠^*** | 2-wk: 2>1  1-m: 1>2  *indicates superiority of the self-regulation over motivation* |
| Matvienko (2001), (50) | 0 | USA | University | 18-26yrs, 0% male | 40 | F2F (group) | I: College course for credit, composed of both lectures and laboratory exercises. Topics included: energy balance and its control by genetic, dietary, and physical activity factors. Classroom exercises included estimation of the BMR, body composition, fat distribution, energy expenditure, and fat use for various physical activities. Laboratory exercises included body composition measurements, serving sizes, food sensory exercises with low- and high-fat foods, and food preparation methods  C: Usual lifestyle | 4-m | 0, 4-m, 16-m | 16-m: 83% | FFQ | Energy intake (kcal/day) | 4-m: 1>2  16-m: 1=2 |
|  |  |  |  |  |  |  |  |  |  |  |  | Energy from fat (%) | 4-m: 1=2  16-m: 1>2 |
|  |  |  |  |  |  |  |  |  |  |  |  | Total fat (g/d) | 4-m: 1=2  16-m: 1=2 |
|  |  |  |  |  |  |  |  |  |  |  |  | Total CHO (g/day) | 4-m: 1>2  16-m: 1=2 |
|  |  |  |  |  |  |  |  |  |  |  |  | Total protein (g/day) | 4-m: 1>2  16-m: 1=2 |
| Meng (2017), (51) | 0 | USA | University | 18-22yrs, 33% male | 338 | F2F (group) | All intervention arms joined a four-person group wherein every group member posted self-tracking of their fruit and vegetable consumption three times a week.  I (arm 1): Plus demographic similar x incremental change  I (arm 2): Plus demographic similar x ideal change  I (arm 3): Plus demographic diverse x incremental change  I (arm 4): Plus demographic diverse x ideal change  C (arm 5): Accessed the webpage and reported self-tracking of their fruit and vegetable consumption three times a week without the presence of group members. | 4-wk | 0, 4-wk | 4-wk: 22% | FFQ | Fruit and Veg (frequency)***^≠^*** | 4-wk: 1>5, 2>5, 3>5, 4>5 |
| Middleton (2014), (52) | 0 | USA | University | 18-19yrs, 0% male | 95 | F2F (group) | I: 4-week, 5-session weight gain prevention program conducted with closed groups. Key components of this program included decreasing caloric intake, increasing physical activity, implementing self-monitoring of weight, and improving self-regulatory skills.  C: Wait list control | 4-wks | 0, 7-wk, 15-wks | 15-wk: 84% | Diet recall (ASA-24) | Energy intake (calories/day) | 7-wk: 1=2, 15-wk: 1=2 |
| Nix (2017), (53) | 0 | USA | University | 18-24yrs, 24% male | 222 | Email | I (arm 1): Sent an email thanking them for their participation and informing them that the USDA recommendation for FV intake is 4-5 cups per day.  I (arm 2): High Norm message: regardless of actual intake, these participants received a message that stated “You are in the 74th-82nd percentile  I (arm 3): Low Norm message: provided with a nearly identical message to those in the High Norm group. However, this group was informed that they were in the 19th-25^th^ percentile of intake, regardless of actual intake.  C (arm 4): Email thanking them for their participation and completed second survey | 1-wk | 0, 1-wk | 1-wk: 75% | NCI fruit and Veg screener | Fruit and Veg intake (cups/day)***^≠^*** | 1-wk: 3>2, 3=4, 3=1, 2=4, 1=4, 1=2 |
| O’Brien (2016), (54) | 0 | USA | University | 18-26yrs, 32% male | 154 | Website + SMS | I (arm 1): Web-based intervention - on-screen, open-response prompts- asked to elaborate on personally valued qualities of hypothetical peer/version of self that has achieved health and fitness. This group was also presented with detailed personalised feedback  I (arm 2): Web-based intervention + daily messages. Same as Arm 1 but with additional text messages over 30 days  C (arm 3): Assessment only | 1-m | 0, 1-m | 1-m: 96% | 7-day diet recall | Fruit (% meeting fruit guidelines)***^≠^*** | 1-m: 1=2=3 |
|  |  |  |  |  |  |  |  |  |  |  |  | Veg (% meeting veg guidelines) | 1-m: 1=3, 2>3 |
| Ohtsuki (2018), (55) | 0 | Japan | University | 18-24yrs, 24% male | 104 | F2F | I: Three educational projects; a lesson on the nutritional significance of vegetable consumption, a tour of an agricultural farm, and learning of cooking skills for vegetable intake. Encouraged to eat 350g Veg a day  C: Usual lifestyle | 6-m | 0, 6-m | 6-m: 94% | Diet History Questionnaire | Vegetables (g/day) | 6-m: 1=2 |
|  |  |  |  |  |  |  |  |  |  |  |  | Green & Yellow veg (g/day) | 6-m: 1>2 |
|  |  |  |  |  |  |  |  |  |  |  |  | Fruit (g/day) | 6-m: 1=2 |
|  |  |  |  |  |  |  |  |  |  |  |  | Fish (g/day) | 6-m: 1=2 |
|  |  |  |  |  |  |  |  |  |  |  |  | Meat (g/day) | 6-m: 1=2 |
|  |  |  |  |  |  |  |  |  |  |  |  | Staple food (g/day) | 6-m: 1=2 |
| Ortega (2006), (56) | 0 | Spain | Community | 20-35yrs, 0% males | 67 | F2F | I: Weight control measures based on increasing the proportional consumption of greens and vegetables and restricting consumption of energy-rich foods.  C: Active control: weight control measures based on increasing the proportional consumption of cereals | 6-wk | 0, 2-wks, 6-wks | 6-wks: 85% | 3-day Food record | Energy intake (kJ/day) | 2-wk: 1=2  6-wk: 1=2 |
|  |  |  |  |  |  |  |  |  |  |  |  | Protein (g/day) | 2-wk: 1=2  6-wk: 1=2 |
|  |  |  |  |  |  |  |  |  |  |  |  | Protein (% energy) | 2-wk: 1=2  6-wk: 1=2 |
|  |  |  |  |  |  |  |  |  |  |  |  | CHO (g/day) | 2-wk: 2>1  6-wk: 1=2 |
|  |  |  |  |  |  |  |  |  |  |  |  | CHO (% energy | 2-wk: 2>1  6-wk: 2>1 |
|  |  |  |  |  |  |  |  |  |  |  |  | Fats (g/day) | 2-wk: 2>1  6-wk: 2=1 |
|  |  |  |  |  |  |  |  |  |  |  |  | Fats (% energy) | 2-wk: 2>1  6-wk: 2>1 |
|  |  |  |  |  |  |  |  |  |  |  |  | Fibre (g/day) | 2-wk: 2>1  6-wk: 2>1 |
|  |  |  |  |  |  |  |  |  |  |  |  | Greens & veg (servings/day) | 2-wk: 1>2  6-wk: 1>2 |
|  |  |  |  |  |  |  |  |  |  |  |  | Fruit (serves/day) | 2-wk: 1=2  6-wk: 1=2 |
|  |  |  |  |  |  |  |  |  |  |  |  | Cereals (serves/day) | 2-wk: 2>1  6-wk: 2>1 |
|  |  |  |  |  |  |  |  |  |  |  |  | HEI score | 2-wk: 2>1  6-wk: 2>1 |
| Park (2008), (57) | 0 | USA | Community | 18-24yrs, 33% male | 160 | Website | I: TTM-tailored treatment adapted from a multimodal (print and telephone) intervention that had successfully increased fruit and vegetable consumption among young adults.  C: Provided action-oriented messages based on standard 5-A-Day nutrition messages. | 2-m | 0, 2-m | 2-m: 87% | Short diet question | Fruit and Veg (serves/day)***^≠^*** | 2-m: 1=2 |
| Pellitteri (2017), (58) | 0 | USA | University | 18-20yrs, 0% male | 37 | Print + F2F | I: 8 week program based on Fit Minded- a theory-based book club  that helps women acquire the tools they need to become more active and stay active for a lifetime + 3 issues of current women’s health magazine  C: Given 3 issues of current women’s health magazine. | 2-m | 0, 2-m | 2-m: 73% | Short diet questions (Youth Wave screener) | SSB (servings/days) | 2-m: 1>2 |
|  |  |  |  |  |  |  |  |  |  |  |  | Fruit (serves/day) | 2-m: 1=2 |
|  |  |  |  |  |  |  |  |  |  |  |  | Veg (serves/day) | 2-m: 1=2 |
|  |  |  |  |  |  |  |  |  |  |  |  | Junk food (servings/day) | 2-m: 1=2 |
| Richards (2006), (59) | 0 | USA | University | 18-24yrs, 29% male | 437 | Print + F2F + Email | I: Four stage-based newsletters, one motivational interview, and an individually tailored e-mail follow-up over a 4-month period to increase fruit and vegetable consumption.  C: Usual lifestyle | 4-m | 0, 4-m | 4-m: 72% | FFQ | Fruit and Veg (serves/day)***^≠^*** | 4-m: 1>2 |
| Rompotis (2014), (60) | 0 | Australia | University | 18-34yrs, 18% male | 161 | SMS + email | I (arm 1): SMS messages consisting of habit framework principles  C (arm 2): SMS message active control fruit and vegetable consumption  C (arm 3): SMS active control general healthy eating  I (arm 4): E-mail messages consisting of habit framework principles  C (arm 5): E-mail message active control fruit and vegetable consumption  C (arm 6): E-mail active control general healthy eating | 2-m | 0, 2-m | 2-m: 45% | Short diet questions | Fruit (serves/day)***^≠^*** | 2-m: 1> 2, 1>3. 4>5, 4>6 |
|  |  |  |  |  |  |  |  |  |  |  |  | Veg (serves/day)***^≠^*** | 2-m: 1=2=3  4=5=6 |
| Sandrick (2017), (61) | 0 | USA | University | 18-30yrs, 32% male | 60 | F2F + SMS | I: Single face-to-face meeting with a health coach to review results of behavioural questionnaires and to set a health behaviour goal for the 8-week study period. The face-to-face meeting was followed by SMS text messages designed to encourage achievement of the behavioural goal.  C: Usual lifestyle | 2-m | 0, 2-m | 2-m: 97% | FFQ | Diet Score***^≠^*** | 2-m: 1=2 |
| Schweitzer (2016) ‘ALIVE’ (62) | 0 | USA | University | 18-20yrs, 31% male | 148 | Website + email | I: eHealth intervention consisted of A Lifestyle Intervention  via Email (ALIVE), an evidence-based Web-based behaviour  Change program. Included weekly tailored and interactive diet and physical activity goals.  C: Non-diet and non-exercise fact sheets | 6-m | 0, 3-m, 6-m | 3-m: 84%  6-m: 72% | FFQ (block) | Fruit and veg (cups/day)***^≠^*** | 3-m:1=2  6-m: 1=2 |
|  |  |  |  |  |  |  |  |  |  |  |  | Saturated fat (% total kcal) | 3-m:1>2  6-m: 1>2 |
|  |  |  |  |  |  |  |  |  |  |  |  | Sugar (% total kcal) | 3-m:1=2  6-m: 1=2 |
| Share (2015), (63) | 0 | Australia | University | 18-30yrs, 0% male | 39 | F2F (group) | I: 12 week lifestyle intervention comprised of three main components (1) physical activity (2) nutrition education, and (3) cognitive behavioural therapy  C: Wait list control | 3-m | 0, 3-m | 3-m: 70% | 3-day diet recall | Energy intake (kJ/day) | 3-m: 1=2 |
|  |  |  |  |  |  |  |  |  |  |  |  | CHO (g/day) | 3-m: 1=2 |
|  |  |  |  |  |  |  |  |  |  |  |  | Protein (g/day) | 3-m: 1=2 |
|  |  |  |  |  |  |  |  |  |  |  |  | Fats (g/day) | 3-m: 1=2 |
| Stephens (2017), (64) | 1 (65) | USA | Community | 18-25yrs, 29% male | 62 | Mobile app + F2F + SMS | I: One-time counselling session + additional counselling (content included on energy balance, nutrient density of foods,  SSB consumption, and physical activity); guided to use lose it! smartphone App + individualised text messages,  C: One time counselling session. | 3-m | 0, 3-m | 3-m: 95% | 24-hr recall (ASA-24) | Energy intake (calories/day) | 3-m: 1=2 |
|  |  |  |  |  |  |  |  |  |  |  |  | Protein (unit not provided) | 3-m: 1=2 |
|  |  |  |  |  |  |  |  |  |  |  |  | CHO (unit not provided) | 3-m: 1=2 |
|  |  |  |  |  |  |  |  |  |  |  |  | Fat (unit not provided) | 3-m: 1=2 |
|  |  |  |  |  |  |  |  |  |  |  |  | Sugar (unit not provided) | 3-m: 1=2 |
|  |  |  |  |  |  |  |  |  |  |  |  | Fibre (unit not provided) | 3-m: 1>2 |
|  |  |  |  |  |  |  |  |  |  |  |  | Water (unit not provided) | 3-m: 1=2 |
|  |  |  |  |  |  |  |  |  |  |  |  | Sodium (unit not provided) | 3-m: 1=2 |
|  |  |  |  |  |  |  |  |  |  |  |  | Sat fat (unit not provided) | 3-m: 1=2 |
|  |  |  |  |  |  |  |  |  |  |  |  | Vegetables (unit not provided) | 3-m: 1=2 |
|  |  |  |  |  |  |  |  |  |  |  |  | Fruit (unit not provided) | 3-m: 1=2 |
|  |  |  |  |  |  |  |  |  |  |  |  | Dairy (unit not provided) | 3-m: 1=2 |
|  |  |  |  |  |  |  |  |  |  |  |  | Added sugars (unit not provided) | 3-m: 1=2 |
| Stice (2013) ‘Healthy Weight 2’ (66) | 1 (67) | USA | University | 17-20yrs, 0% male | 398 | F2F (group) | I: Weekly 1 hour group sessions which promoted lasting healthy improvements to dietary intake and physical activity and nutrition science health behaviour change principles  C: Educational brochure | 1-m | 0, 1-m, 6-m, 12-m, 24-m | 1-m: 96%  6-m: 94%  12-m: 94%  24-m: 93% | FFQ (Block) | Energy intake (Kcal/day) | 1-m: 1=2  6-m: 1=2  12-m: 1=2  24-m: 1=2 |
| Svetkey (2015) ‘CITY’ (68) | 3 (69-71) | USA | Community | 18-35yrs, 30% male | 365 | F2F (group), mobile app | I (arm 1): Six weekly group sessions followed by a monthly one-on-one coaching call from an interventionist for the remainder of the 24 month intervention. Cell phone app used for self-monitoring with feedback provided by the interventionist  I (arm 2): Cell phone application (app) many similar behavioural strategies that are stressed in arm 1 including self-monitoring, problem solving, relapse prevention, stimulus control, goal- setting, action-planning and progress-monitoring toward goals. The app also delivers tailored and individualized feedback.  C (arm 3): Given health education materials | 24-m | 0, 6-m, 12-m, 24-m | 6-m: 92%  12-m: 89%  24-m: 86% | 24-hr recall | Healthy Index Score | 6-m: 1=2=3  12-m: 1=2=3  24-m: 2<3, 1=2, 1=3 |
| Tavakoli (2016), (72) | 0 | Iran | Military University | 18-32yrs, 100% male | 280 | F2F (group) | I: The health belief model (HBM) education- included a combination of lectures, question-and answer sessions, and brainstorming. The content included an introduction to nutritional concepts, the food pyramid, the role of nutrition in lifestyle, and information about  Nutrition-related diseases.  C: No education | 1-m | 0, 1-m | 1-m: 86% | 19-item FFQ | Diet behaviour score***^≠^*** | 1-m: 1>2 |
| Uglem (2013), (73) | 0 | Norway | Military | 18-26yrs, 100% male | 917 | Print + F2F | I: Change in food environment particularly increased availability of vegetables and semi-wholegrain breads, in addition recruits received nutrition information.  C: No intervention | 5-m | 0, 5-m | 5-m: 61% | Food diary | Vegetables, (g/day) ***^≠^*** | 5-m: 1>2 |
|  |  |  |  |  |  |  |  |  |  |  |  | Fruit (g/day) | 5-m: 1>2 |
|  |  |  |  |  |  |  |  |  |  |  |  | Potatoes (g/day) | 5-m: 1>2 |
|  |  |  |  |  |  |  |  |  |  |  |  | Whole grain bread (g/day) | 5-m: 1>2 |
|  |  |  |  |  |  |  |  |  |  |  |  | Juice (g/day) | 5-m: 1>2 |
|  |  |  |  |  |  |  |  |  |  |  |  | Whole grain bread (g/day) | 5-m: 1=2 |
| Werch (2010) ‘Project fitness’ (74) | 2 (75, 76) | USA | University | 18-21yrs, 41% male | 299 | F2F (individual) | I: One-on-one consultation using a consultation protocol with scripted messages which lasted about 25 minutes. At the conclusion of the consult, participants received a one-page goal plan.  C: Usual lifestyle | 3-m | 0, 3-m, 12-m | 3-m: 95%  12-m: 77% | Short diet questions | Fruit and Veg (serves/day) | 3-m: 1=2  *Diet outcomes only reported at 3-m* |
|  |  |  |  |  |  |  |  |  |  |  |  | CHO (frequency) | 3-m: 1=2 |
|  |  |  |  |  |  |  |  |  |  |  |  | Fats (frequency) | 3-m: 1=2 |
| Williams (2002), (77) | 0 | USA | University | 20-25yrs, 100% male | 45 | F2F (individual) | I (arm 1): Nutrition counselling and measurement of serum cholesterol  I (arm 2): Nutrition counselling only  I (arm 3): Measurement of serum cholesterol only  C (arm 4): Usual lifestyle | 6-wk | 0, 6-wks | NR | 24-hr recall and 2-day food record | % kcal from fat ***^≠^*** | 6-wk: 1>4, 1=2, 1=3, 2=3, 2=4, 3=4 |
| Zhang (2012) (78) | 0 | UK | University | 18-24yrs, NR | 173 | Print | I (arm 1): Motivational intervention (PMT): leaflet containing  information from the websites of Diabetes UK, the NHS, the  American Diabetes Association, and WHO, designed to target  PMT variables in relation to type 2 diabetes  I (arm 2): Volitional intervention (APCP) completed action and coping planning sheets i.e., ‘Where’, ‘When’, ‘How’, and ‘With whom’ will you eat healthy.  I (arm 3): Combined (PMT & ACPC)  C (arm4): Usual lifestyle | 2-wks | 0, 2-wks, 6-wks | 6-wks: 48% | FFQ | Fruit & Veg (portions/day)***^≠^*** | 6:wk 1=2=3=4  *Only reported change from 2-wk to 6-wk* |
|  |  |  |  |  |  |  |  |  |  |  |  | % fat intake | 6:wk 1=2=3=4  *Only reported change from 2-wk to 6-wk* |

***^≠^*** Primary outcome of the study

*1 = 2: no differences between groups 1 & 2 (*P* > 0.05); 1 > 2: group 1 had significantly greater improvements on outcome than group 2 (*P* ≤ 0.05).

**Abbreviations:** SSB = Sugar Sweetened Beverages; F2F = Face to Face; ED-NP = Energy Dense Nutrient Poor; CHO = Carbohydrate

**References**

1. Allman-Farinelli M, Partridge SR, McGeechan K, Balestracci K, Hebden L, Wong A, et al. A Mobile Health Lifestyle Program for Prevention of Weight Gain in Young Adults (TXT2BFiT): Nine-Month Outcomes of a Randomized Controlled Trial. Jmir Mhealth and Uhealth. 2016;4(2):408-19.

2. Partridge SR, Allman-Farinelli M, McGeechan K, Balestracci K, Wong AT, Hebden L, et al. Process evaluation of TXT2BFiT: a multi-component mHealth randomised controlled trial to prevent weight gain in young adults. International Journal of Behavioral Nutrition & Physical Activity. 2016;13:7.

3. Partridge SR, Balestracci K, Wong ATY, Hebden L, McGeechan K, Denney-Wilson E, et al. Effective Strategies to Recruit Young Adults Into the TXT2BFiT mHealth Randomized Controlled Trial for Weight Gain Prevention. Jmir Research Protocols. 2015;4(2).

4. Partridge SR, McGeechan K, Bauman A, Phongsavan P, Allman-Farinelli M. Improved eating behaviours mediate weight gain prevention of young adults: Moderation and mediation results of a randomised controlled trial of TXT2BFiT, mHealth program. The International Journal of Behavioral Nutrition and Physical Activity Vol 13 2016, ArtID 44. 2016;13.

5. Partridge SR, McGeechan K, Bauman A, Phongsavan P, Allman-Farinelli M. Improved confidence in performing nutrition and physical activity behaviours mediates behavioural change in young adults: Mediation results of a randomised controlled mHealth intervention. Appetite. 2017;108:425-33.

6. Partridge SR, McGeechan K, Hebden L, Balestracci K, Wong ATY, Denney-Wilson E, et al. Effectiveness of a mHealth Lifestyle Program With Telephone Support (TXT2BFiT) to Prevent Unhealthy Weight Gain in Young Adults: Randomized Controlled Trial. Jmir Mhealth and Uhealth. 2015;3(2).

7. Hebden L, Balestracci K, McGeechan K, Denney-Wilson E, Harris M, Bauman A, et al. 'TXT2BFiT' a mobile phone-based healthy lifestyle program for preventing unhealthy weight gain in young adults: study protocol for a randomized controlled trial. Trials [Electronic Resource]. 2013;14:75.

8. Annesi JJ, Howton A, Johnson PH, Porter KJ. Pilot testing a cognitive-behavioral protocol on psychosocial predictors of exercise, nutrition, weight, and body satisfaction changes in a college-level health-related fitness course. Journal of American College Health. 2015;63(4):268-78.

9. Ashton LM, Morgan PJ, Hutchesson MJ, Rollo ME, Collins CE. Feasibility and preliminary efficacy of the 'HEYMAN' healthy lifestyle program for young men: a pilot randomised controlled trial. Nutrition Journal. 2017;16(1):1-17.

10. Brookie KL, Mainvil LA, Carr AC, Vissers MC, Conner TS. The development and effectiveness of an ecological momentary intervention to increase daily fruit and vegetable consumption in low-consuming young adults. Appetite. 2017;108:32-41.

11. Buscemi J, Yurasek AM, Dennhardt AA, Martens MP, Murphy JG. A randomized trial of a brief intervention for obesity in college students. Clinical Obesity. 2011;1(4-6):131-40.

12. Chang MW, Nitzke S, Brown R. Design and outcomes of a Mothers In Motion behavioral intervention pilot study. Journal of Nutrition Education & Behavior. 2010;42(3 Suppl):S11-21.

13. Chang MW, Brown R, Nitzke S. Participant recruitment and retention in a pilot program to prevent weight gain in low-income overweight and obese mothers. BMC Public Health. 2009;9:424.

14. Chapman J, Armitage CJ, Norman P. Comparing implementation intention interventions in relation to young adults' intake of fruit and vegetables. Psychology & Health. 2009;24(3):317-32.

15. Do M, Kattelmann K, Boeckner L, Greene G, White A, Hoerr S, et al. Low-income young adults report increased variety in fruit and vegetable intake after a stage-tailored intervention. Nutrition Research. 2008;28(8):517-22.

16. Nitzke S, Kritsch K, Boeckner L, Greene G, Hoerr S, Horacek T, et al. A stage-tailored multi-modal intervention increases fruit and vegetable intakes of low-income young adults. American Journal of Health Promotion. 2007;22(1):6-14.

17. Eiben G, Lissner L. Health Hunters--an intervention to prevent overweight and obesity in young high-risk women2006; 30(4):[691-6 pp.]. Available from: <http://onlinelibrary.wiley.com/o/cochrane/clcentral/articles/440/CN-00561440/frame.html>.

18. Franko DL, Cousineau TM, Trant M, Green TC, Rancourt D, Thompson D, et al. Motivation, self-efficacy, physical activity and nutrition in college students: Randomized controlled trial of an internet-based education program. Preventive Medicine: An International Journal Devoted to Practice and Theory. 2008;47(4):369-77.

19. Franko DL, Jenkins A, Rodgers RF. Toward Reducing Risk for Eating Disorders and Obesity in Latina College Women. Journal of Counseling and Development. 2012;90(3):298-307.

20. Goodman S, Morrongiello B, Meckling K. A randomized, controlled trial evaluating the efficacy of an online intervention targeting vitamin D intake, knowledge and status among young adults. International Journal of Behavioral Nutrition & Physical Activity. 2016;13(1):116.

21. Gow RW, Trace SE, Mazzeo SE. Preventing weight gain in first year college students: An online intervention to prevent the "freshman fifteen.". Eating Behaviors. 2010;11(1):33-9.

22. Greene GW, White AA, Hoerr SL, Lohse B, Schembre SM, Riebe D, et al. Impact of an Online Healthful Eating and Physical Activity Program for College Students. American Journal of Health Promotion. 2012;27(2):E47-E58.

23. Dour CA, Horacek TM, Schembre SM, Lohse B, Hoerr S, Kattelmann K, et al. Process evaluation of Project WebHealth: a nondieting Web-based intervention for obesity prevention in college students. Journal of Nutrition Education & Behavior. 2013;45(4):288-95.

24. Hebden L, Cook A, van der Ploeg HP, King L, Bauman A, Allman-Farinelli M. A mobile health intervention for weight management among young adults: a pilot randomised controlled trial. Journal of Human Nutrition & Dietetics. 2014;27(4):322-32.

25. Heeren GA, Jemmott JB, Marange CS, Rumosa Gwaze A, Batidzirai JM, Ngwane Z, et al. Health-Promotion Intervention Increases Self-Reported Physical Activity in Sub-Saharan African University Students: A Randomized Controlled Pilot Study. Behavioral Medicine. 2017:1-9.

26. Hivert M, Langlois M, Berard P, Cuerrier J, Carpentier A. Prevention of weight gain in young adults through a seminar-based intervention program. International Journal of Obesity. 2007;31(8):1262-9.

27. Hutchesson MJ, Callister R, Morgan PJ, Pranata I, Clarke ED, Skinner G, et al. A Targeted and Tailored eHealth Weight Loss Program for Young Women: The Be Positive Be Healthe Randomized Controlled Trial. Healthcare. 2018;6(2).

28. Jakicic JM, Davis KK, Rogers RJ, King WC, Marcus MD, Helsel D, et al. Effect of wearable technology combined with a lifestyle intervention on long-term weight loss: The IDEA randomized clinical trial. JAMA - Journal of the American Medical Association. 2016;316(11):1161-71.

29. Jakicic JM, King WC, Marcus MD, Davis KK, Helsel D, Rickman AD, et al. Short-term weight loss with diet and physical activity in young adults: The IDEA study. Obesity (19307381). 2015;23(12):2385-97.

30. Jung ME, Martin Ginis KA, Phillips SM, Lordon CD. Increasing calcium intake in young women through gain-framed, targeted messages: A randomised controlled trial. Psychology & Health. 2011;26(5):531-47.

31. Kattelmann KK, Bredbenner CB, White AA, Greene GW, Hoerr SL, Kidd T, et al. The effects of Young Adults Eating and Active for Health (YEAH): A theory-based web-delivered intervention. Journal of Nutrition Education and Behavior. 2014;46(6):S27-S41.

32. Brown O, Quick V, Colby S, Greene G, Horacek TM, Hoerr S, et al. Recruitment lessons learned from a tailored web-based health intervention project Y.E.A.H. (young adults eating and active for health). Health Education. 2015;115(5):470-9.

33. Kattelmann KK, White AA, Greene GW, Byrd-Bredbenner C, Hoerr SL, Horacek TM, et al. Development of Young Adults Eating and Active for Health (YEAH) internet-based intervention via a community-based participatory research model. Journal of nutrition education and behavior. 2014;46(2):S10-S25.

34. Kendzierski D, Ritter RL, Stump TK, Anglin CL. The effectiveness of an implementation intentions intervention for fruit and vegetable consumption as moderated by self-schema status. Appetite. 2015;95:228-38.

35. Kerr DA, Harray AJ, Pollard CM, Dhaliwal SS, Delp EJ, Howat PA, et al. The connecting health and technology study: A 6-month randomized controlled trial to improve nutrition behaviours using a mobile food record and text messaging support in young adults. The International Journal of Behavioral Nutrition and Physical Activity Vol 13 2016, ArtID 52. 2016;13.

36. Kerr DA, Pollard CM, Howat P, Delp EJ, Pickering M, Kerr KR, et al. Connecting Health and Technology (CHAT): protocol of a randomized controlled trial to improve nutrition behaviours using mobile devices and tailored text messaging in young adults. BMC Public Health. 2012;12:477.

37. Knauper B, McCollam A, Rosen-Brown A, Lacaille J, Kelso E, Roseman M. Fruitful plans: Adding targeted mental imagery to implementation intentions increases fruit consumption. Psychology & Health. 2011;26(5):601-17.

38. Kothe EJ, Mullan BA, Butow P. Promoting fruit and vegetable consumption. Testing an intervention based on the theory of planned behaviour. Appetite. 2012;58(3):997-1004.

39. Kothe EJ, Mullan BA. A randomised controlled trial of a theory of planned behaviour to increase fruit and vegetable consumption. Fresh Facts. Appetite. 2014;78:68-75.

40. Kothe EJ, Mullan BA. Factors affecting acceptability of an email-based intervention to increase fruit and vegetable consumption. BMC Public Health. 2014;14:1020.

41. Kreausukon P, Gellert P, Lippke S, Schwarzer R. Planning and self-efficacy can increase fruit and vegetable consumption: a randomized controlled trial. Journal of Behavioral Medicine. 2012;35(4):443-51.

42. Kypri K, McAnally HM. Randomized controlled trial of a web-based primary care intervention for multiple health risk behaviors. Preventive Medicine. 2005;41(3-4):761-6.

43. LaChausse RG. My student body: Effects of an internet-based prevention program to decrease obesity among college students. Journal of American College Health. 2012;60(4):324-30.

44. Laska MN, Lytle LA, Nanney MS, Moe SG, Linde JA, Hannan PJ. Results of a 2-year randomized, controlled obesity prevention trial: Effects on diet, activity and sleep behaviors in an at-risk young adult population. Preventive Medicine: An International Journal Devoted to Practice and Theory. 2016;89:230-6.

45. Laska MN, Sevcik SM, Moe SG, Petrich CA, Nanney MS, Linde JA, et al. A 2-year young adult obesity prevention trial in the US: Process evaluation results. Health Promotion International. 2016;31(4):793-800.

46. Lytle LA, Laska MN, Linde JA, Moe SG, Nanney MS, Hannan PJ, et al. Weight-gain reduction among 2-year college students: The CHOICES RCT. American Journal of Preventive Medicine. 2017;52(2):183-91.

47. Lytle LA, Moe SG, Nanney MS, Laska MN, Linde JA, Petrich CA, et al. Designing a Weight Gain Prevention Trial for Young Adults: The CHOICES Study. American Journal of Health Education. 2014;45(2):67-75.

48. Moe SG, Lytle LA, Nanney MS, Linde JA, Laska MN. Recruiting and retaining young adults in a weight gain prevention trial: Lessons learned from the CHOICES study. Clinical Trials. 2016;13(2):205-13.

49. Lhakhang P, Godinho C, Knoll N, Schwarzer R. A brief intervention increases fruit and vegetable intake. A comparison of two intervention sequences. Appetite. 2014;82:103-10.

50. Matvienko O, Lewis DS, Schafer E. A college nutrition science course as an intervention to prevent weight gain in female college freshmen. Journal of Nutrition Education. 2001;33(2):95-101.

51. Meng J, Peng W, Shin SY, Chung M. Online Self-Tracking Groups to Increase Fruit and Vegetable Intake: A Small-Scale Study on Mechanisms of Group Effect on Behavior Change. Journal of Medical Internet Research. 2017;19(3):e63.

52. Middleton KR, Perri MG. A Randomized Trial Investigating the Effect of a Brief Lifestyle Intervention on Freshman-Year Weight Gain. Journal of American College Health. 2014;62(2):101-9.

53. Nix E, Wengreen HJ. Social approval bias in self-reported fruit and vegetable intake after presentation of a normative message in college students. Appetite. 2017;116:552-8.

54. O'Brien LM, Palfai TP. Efficacy of a brief web-based intervention with and without SMS to enhance healthy eating behaviors among university students. Eating Behaviors. 2016;23:104-9.

55. Ohtsuki M, Shibata K, Fukuwatari T, Sasaki Y, Nakai K. Randomized controlled trial of educational intervention to increase consumption of vegetables by Japanese university students. Health Education. 2018;118(4):290-303.

56. Ortega RM, Rodriguez-Rodriguez E, Aparicio A, Marin-Arias LI, Lopez-Sobaler AM. Responses to two weight-loss programs based on approximating the diet to the ideal: Differences associated with increased cereal or vegetable consumption. International Journal for Vitamin and Nutrition Research. 2006;76(6):367-76.

57. Park A, Nitzke S, Kritsch K, Kattelmann K, White A, Boeckner L, et al. Internet-based interventions have potential to affect short-term mediators and indicators of dietary behavior of young adults. Journal of Nutrition Education and Behavior. 2008;40(5):288-97.

58. Pellitteri K, Huberty J, Ehlers D, Bruening M. Fit Minded College Edition Pilot Study: Can a Magazine-Based Discussion Group Improve Physical Activity in Female College Freshmen? Journal of Public Health Management & Practice. 2017;23(1):e10-e9.

59. Richards A, Kattelmann KK, Ren C. Motivating 18- to 24-year-olds to increase their fruit and vegetable consumption. Journal of the American Dietetic Association. 2006;106(9):1405-11.

60. Rompotis CJ, Grove JR, Byrne SM. Benefits of habit-based informational interventions: a randomised controlled trial of fruit and vegetable consumption. Australian & New Zealand Journal of Public Health. 2014;38(3):247-52.

61. Sandrick J, Tracy D, Eliasson A, Roth A, Bartel J, Simko M, et al. Effect of a Counseling Session Bolstered by Text Messaging on Self-Selected Health Behaviors in College Students: A Preliminary Randomized Controlled Trial. Jmir Mhealth and Uhealth. 2017;5(5).

62. Schweitzer AL, Ross JT, Klein CJ, Lei KY, Mackey ER. An Electronic Wellness Program to Improve Diet and Exercise in College Students: A Pilot Study. Jmir Research Protocols. 2016;5(1).

63. Share BL, Naughton GA, Obert P, Peat JK, Aumand EA, Kemp JG. Effects of a Multi-Disciplinary Lifestyle Intervention on Cardiometabolic Risk Factors in Young Women with Abdominal Obesity: A Randomised Controlled Trial. PLoS ONE [Electronic Resource]. 2015;10(6):e0130270.

64. Stephens JD, Yager AM, Allen J. Smartphone technology and text messaging for weight loss in young adults: A randomized controlled trial. Journal of Cardiovascular Nursing. 2017;32(1):39-46.

65. Stephens JD, Althouse A, Tan A, Melnyk BM. The Role of Race and Gender in Nutrition Habits and Self-Efficacy: Results from the Young Adult Weight Loss Study. J Obes. 2017;2017:5980698.

66. Stice E, Rohde P, Shaw H, Marti C. Efficacy trial of a selective prevention program targeting both eating disorders and obesity among female college students: 1- and 2-year follow-up effects. Journal of Consulting and Clinical Psychology. 2013;81(1):183-9.

67. Stice E, Rohde P, Shaw H, Marti CN. Efficacy trial of a selective prevention program targeting both eating disorder symptoms and unhealthy weight gain among female college students. Journal of Consulting & Clinical Psychology. 2012;80(1):164-70.

68. Svetkey LP, Batch BC, Lin P-H, Intille SS, Corsino L, Tyson CC, et al. Cell Phone Intervention for You (CITY): A randomized, controlled trial of behavioral weight loss intervention for young adults using mobile technology. Obesity. 2015;23(11):2133-41.

69. Batch BC, Tyson C, Bagwell J, Corsino L, Intille S, Lin PH, et al. Weight loss intervention for young adults using mobile technology: design and rationale of a randomized controlled trial - Cell Phone Intervention for You (CITY).[Erratum appears in Contemp Clin Trials. 2014 Nov;39(2):351]. Contemporary Clinical Trials. 2014;37(2):333-41.

70. Corsino L, Lin PH, Batch BC, Intille S, Grambow SC, Bosworth HB, et al. Recruiting young adults into a weight loss trial: report of protocol development and recruitment results. Contemporary Clinical Trials. 2013;35(2):1-7.

71. Lin PH, Intille S, Bennett G, Bosworth HB, Corsino L, Voils C, et al. Adaptive intervention design in mobile health: Intervention design and development in the Cell Phone Intervention for You trial. Clinical Trials. 2015;12(6):634-45.

72. Tavakoli HR, Dini-Talatappeh H, Rahmati-Najarkolaei F, Fesharaki MG. Efficacy of HBM-Based Dietary Education Intervention on Knowledge, Attitude, and Behavior in Medical Students. Iranian Red Crescent Medical Journal. 2016;18(11).

73. Uglem S, Stea TH, Kjollesdal MKR, Frolich W, Wandel M. A nutrition intervention with a main focus on vegetables and bread consumption among young men in the Norwegian National Guard. Food & Nutrition Research. 2013;57.

74. Werch CE, Moore MJ, Bian H, DiClemente CC, Huang IC, Ames SC, et al. Are effects from a brief multiple behavior intervention for college students sustained over time? Preventive Medicine. 2010;50(1-2):30-4.

75. Werch CE, Bian H, Moore MJ, Ames S, DiClemente CC, Weiler RM. Brief multiple behavior interventions in a college student health care clinic. Journal of Adolescent Health. 2007;41(6):577-85.

76. Werch CE, Moore MJ, Bian H, DiClemente CC, Ames SC, Weiler RM, et al. Efficacy of a brief image-based multiple-behavior intervention for college students. Annals of Behavioral Medicine. 2008;36(2):149-57.

77. Williams DR, Lewis NM. Effectiveness of nutrition counseling in young adult males. Nutrition Research. 2002;22(8):911-7.

78. Zhang Y, Cooke R. Using a combined motivational and volitional intervention to promote exercise and healthy dietary behaviour among undergraduates. Diabetes Research & Clinical Practice. 2012;95(2):215-23.
