# Supplementary figures and images for: Effectiveness of Interventions and Behaviour Change Techniques for Improving Dietary Intake in Young Adults: A Systematic Review and Meta-Analysis of RCTs
Source: Nutrients. 2019 Apr 11;11(4):825. doi: 10.3390/nu11040825 (PMC6520715; doi:10.3390/nu11040825)

Figure S1: Funnel Plot vs Standard Error TEI

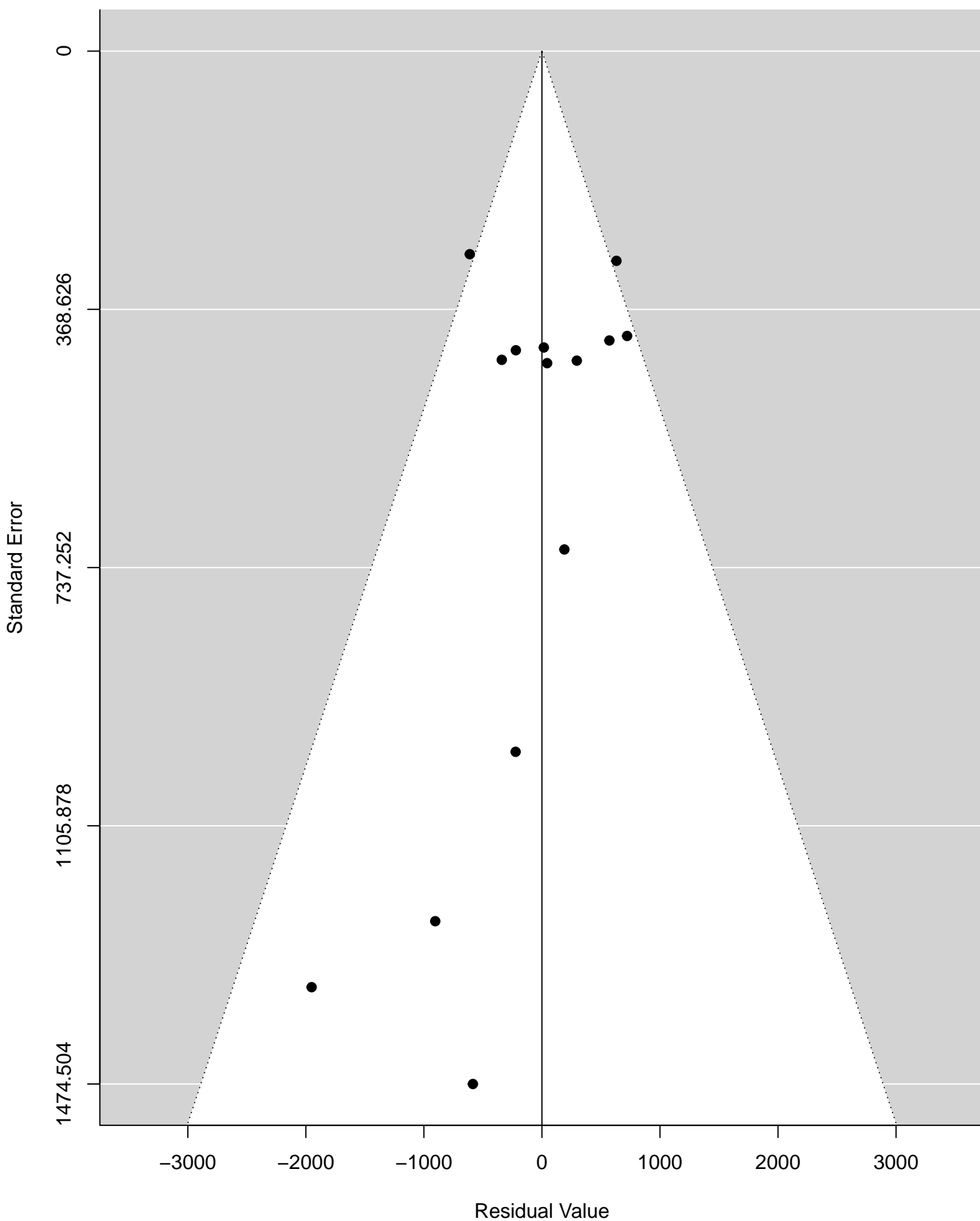

Supplement: Supplementary file 1 [file nutrients-11-00825-s001.zip › Figure S1 Funnel Plot TEI.pdf]

Figure S2: Plots of the means for effects TEI

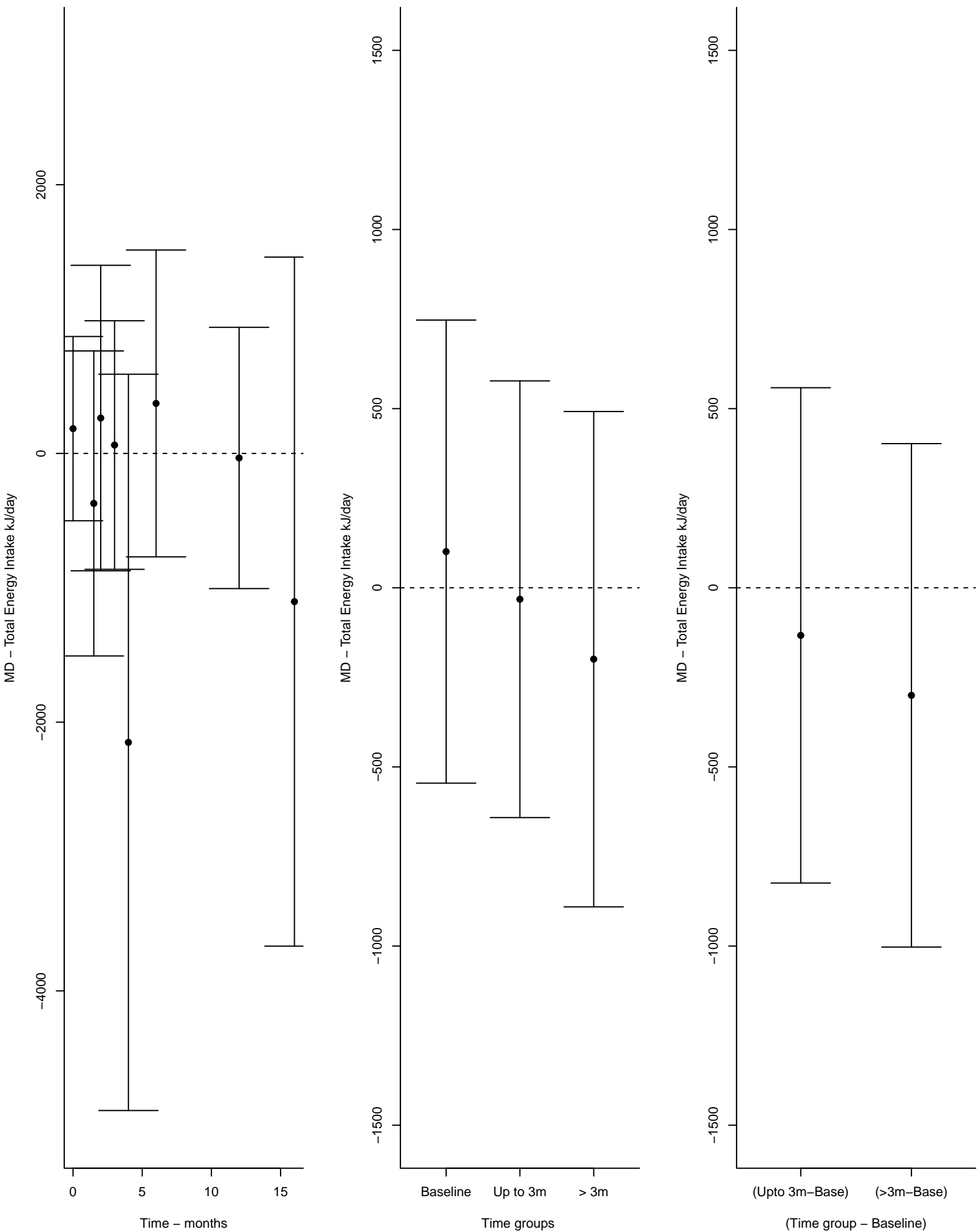

Supplement: Supplementary file 1 [file nutrients-11-00825-s001.zip › Figure S2 Plots of the means for effects TEI.pdf]

Figure S3: Residuals from final model

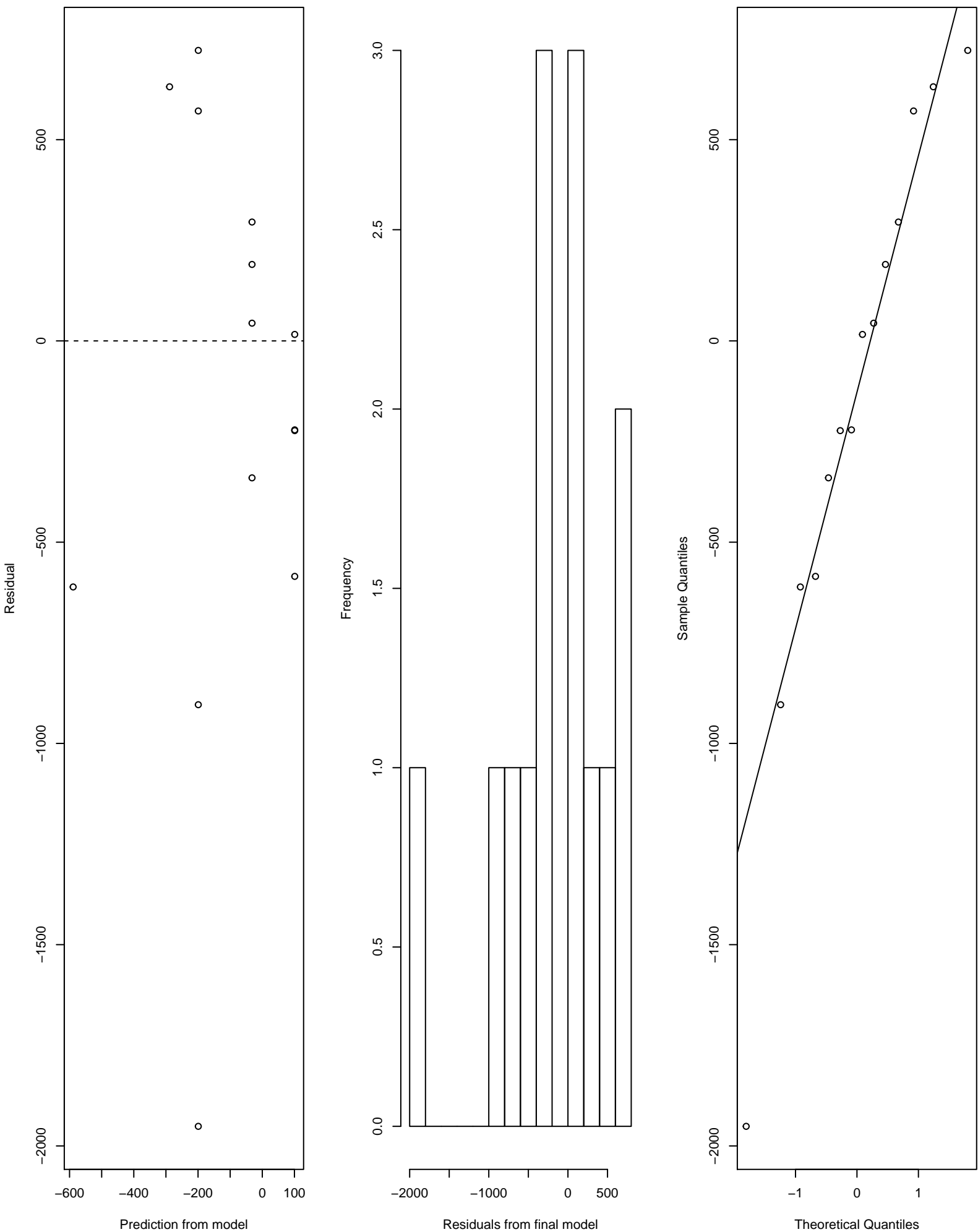

Supplement: Supplementary file 1 [file nutrients-11-00825-s001.zip › Figure S3 Model Diagnostics TEI.pdf]

**Figure S4: Mean Difference – Total Energy Intake kJ/day**

Study/Treatment/Months

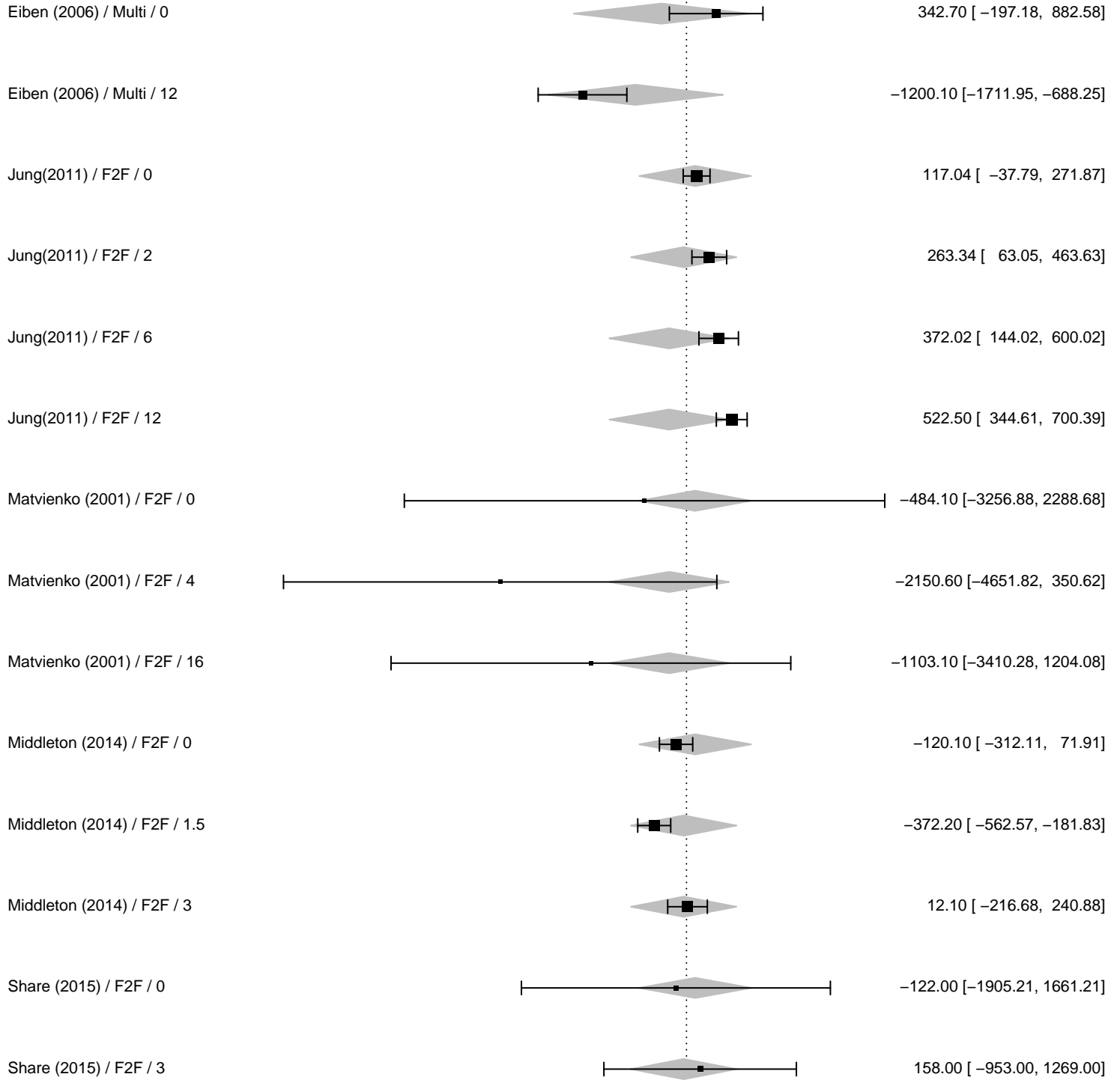

Mean Difference

Supplement: Supplementary file 1 [file nutrients-11-00825-s001.zip › Figure S4 forest plot TEI.pdf]

Figure S5: Funnel Plot vs Standard Error

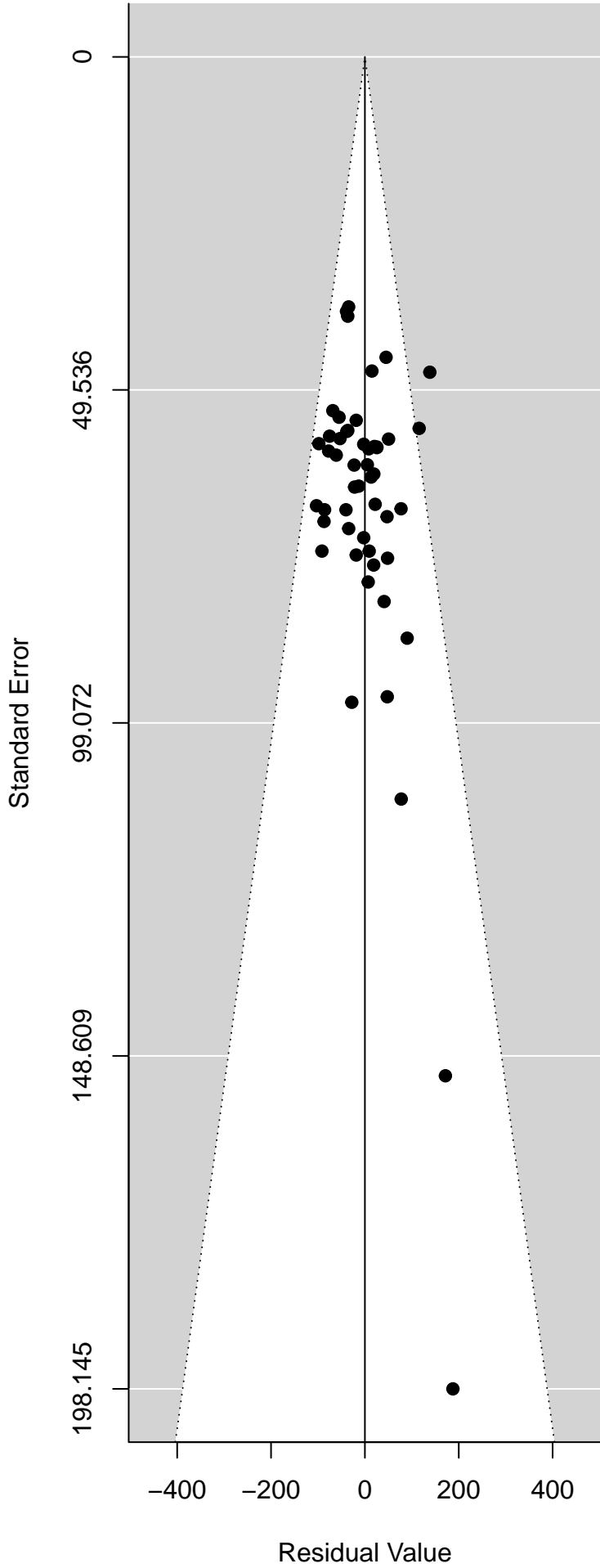

Funnel Plot – removed Chang (2010)

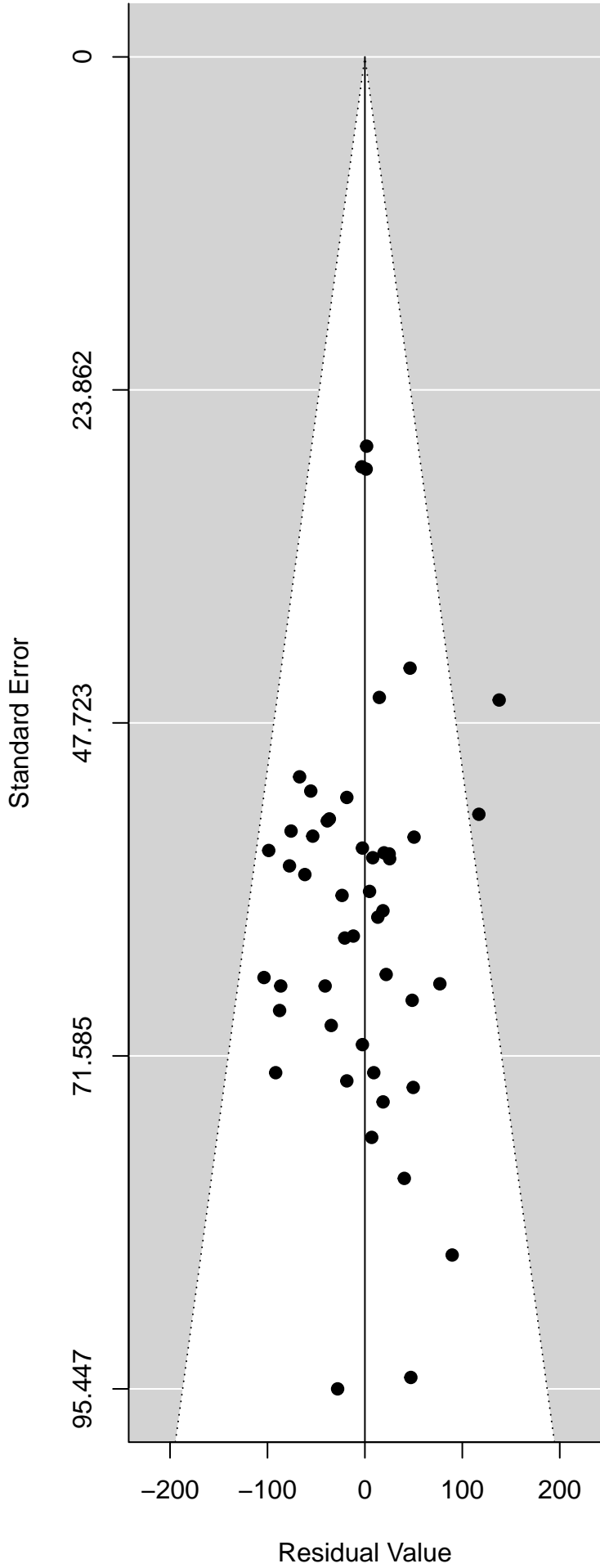

Supplement: Supplementary file 1 [file nutrients-11-00825-s001.zip › Figure S5 Funnel Plot F&V.pdf]

**Figure S6: Plots of the means for effects F&V**

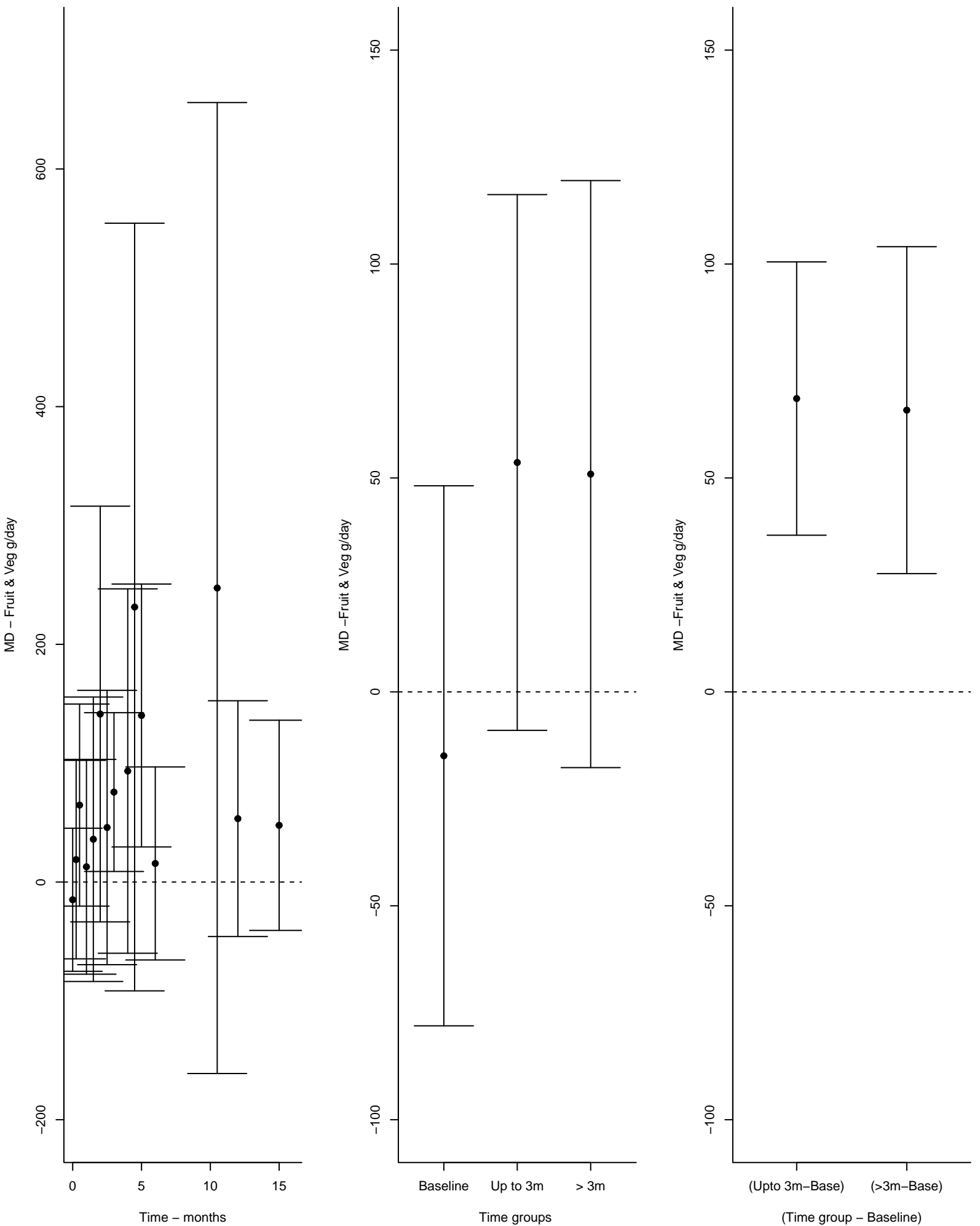

Supplement: Supplementary file 1 [file nutrients-11-00825-s001.zip › Figure S6 Plots of the means for effects F&V.pdf]

Figure S7: Residuals from final model

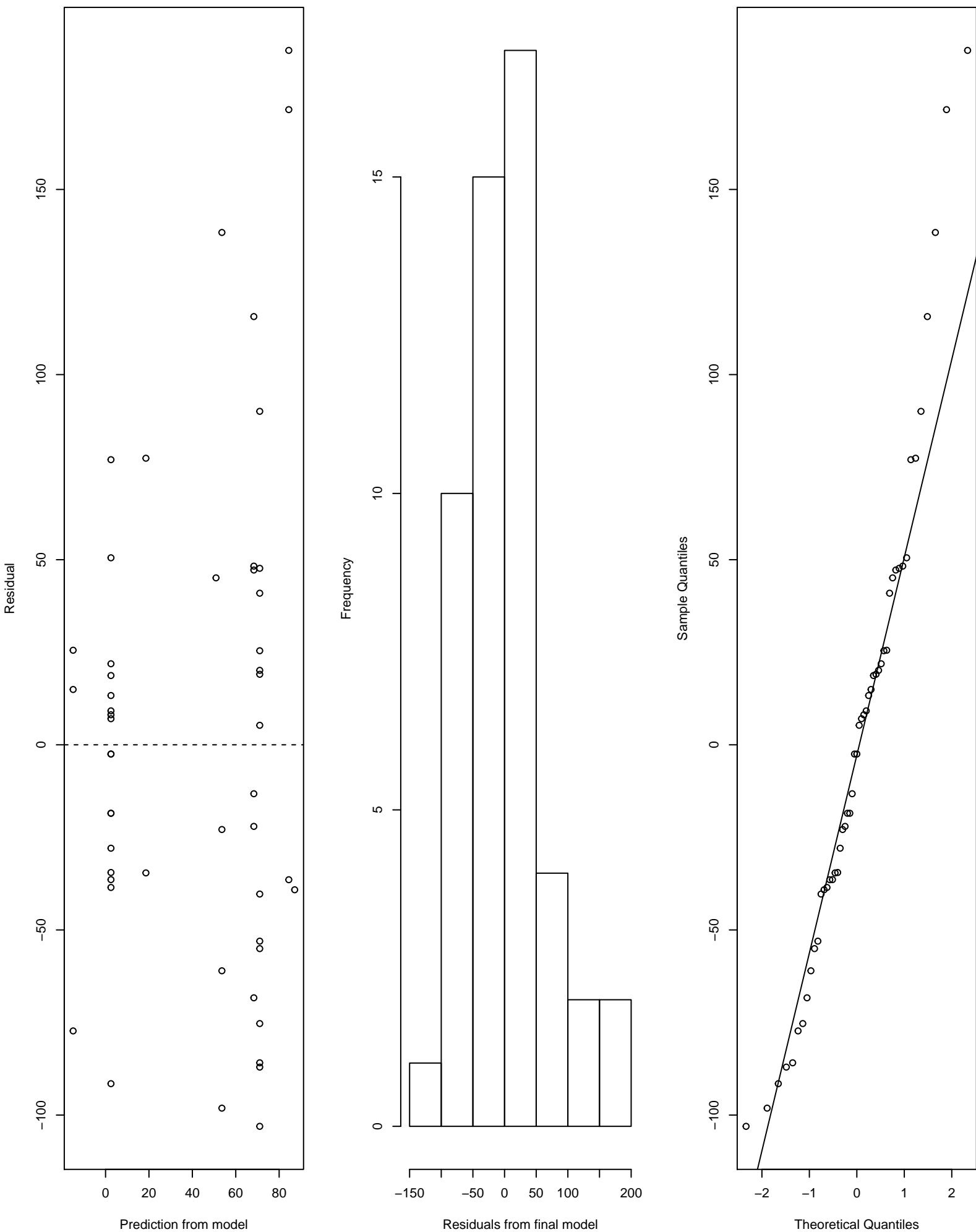

Supplement: Supplementary file 1 [file nutrients-11-00825-s001.zip › Figure S7 Model Diagnostics F&V.pdf]

# Figure S8: Mean Difference – Fruit & Veg g/day

Study/Treatment/Months

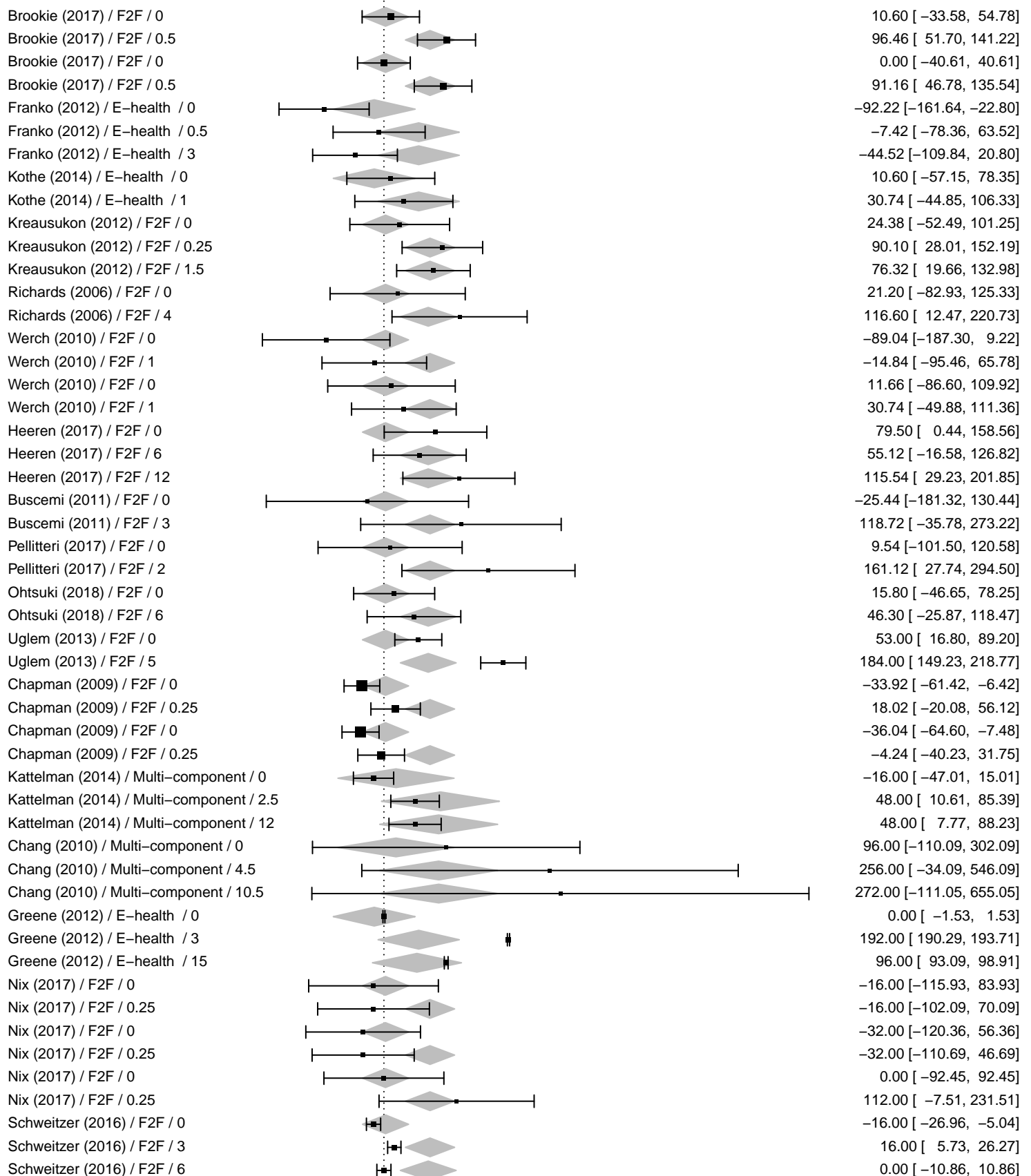

-300

-50

200

450

700

Mean Difference

Supplement: Supplementary file 1 [file nutrients-11-00825-s001.zip › Figure S8 forest plot F&V.pdf]
